# Clinical and Microbiological Outcomes of Critically Ill Patients With Monomicrobial Versus Polymicrobial Bacterial Bloodstream Infections: A Retrospective Cohort Study

**DOI:** 10.1093/ofid/ofaf591

**Published:** 2025-09-24

**Authors:** Anselm Jorda, Felix Bergmann, Marlene Prager, Katarina Kumpf, Georg Gelbenegger, Markus Zeitlinger

**Affiliations:** Department of Clinical Pharmacology, Medical University of Vienna, Vienna, Austria; Department of Clinical Pharmacology, Medical University of Vienna, Vienna, Austria; Department of Clinical Pharmacology, Medical University of Vienna, Vienna, Austria; IT Systems and Communications, Medical University of Vienna, Vienna, Austria; Department of Clinical Pharmacology, Medical University of Vienna, Vienna, Austria; Department of Clinical Pharmacology, Medical University of Vienna, Vienna, Austria

**Keywords:** bacteremia, BSI, ICU, infectiology, sepsis

## Abstract

**Background:**

The impact of polymicrobial bloodstream infections in critically ill patients is uncertain.

**Methods:**

This observational cohort study included patients admitted to the ICU for ≥3d. We compared patients with monomicrobial versus polymicrobial (ie, ≥1 positive blood culture with ≥2 isolates) bloodstream infections. Inverse-probability weighting and covariate-adjusted generalized linear regression models were used to calculate adjusted differences in risks and means. The primary outcome was 90-day all-cause mortality from index culture. Secondary outcomes included 30-day all-cause mortality, microbiological failure, ECMO at 30 days, ARDS at 7 days, fever at 7 days, length of ICU and hospital stay. A separate analysis was performed excluding common skin commensals.

**Results:**

Between 03/2014 and 03/2024, 3197 patients were included in the overall and 1669 in the common commensal-free cohort. In the overall cohort, 90-day mortality occurred in 749 (28.3%, adjusted 28.3%) of 2648 patients in the monomicrobial group and 164 (29.9%, adjusted 31.1%) of 549 patients in the polymicrobial group (adjusted risk difference, 2.84% [95%CI, −1.19–6.88]). All-cause 30-day mortality, microbiological failure (adjusted, 4.67% vs 6.56%), initiation of ECMO (adjusted, 3.65% vs 2.99%), ARDS (adjusted, 17.4% vs 15.7%), fever (adjusted, 19.1% vs 20.4%), length of ICU stay (adjusted means, 19.6 vs 19.8 days), and length of hospital stay (adjusted means, 42.6 vs 45.2 days) did not differ significantly between the groups. Outcomes did not differ in the common commensal-free cohort.

**Conclusions:**

Clinical and microbiological outcomes were comparable between critically ill patients with monomicrobial and polymicrobial bloodstream infections, regardless of whether suspected contaminants were excluded.

Bloodstream infections are a frequent and serious complication in critically ill patients associated with considerable morbidity, prolonged intensive care unit (ICU) stay, high mortality rates, and increased healthcare costs [1]. Polymicrobial bloodstream infections, defined as the presence of two or more distinct isolates of pathogens within the same blood culture are commonly perceived as indicators of severe infection or failure to control the source of infection [2]. As such, they are often assumed to be associated with worse clinical outcomes than monomicrobial infections. Despite this common belief, evidence regarding the prognostic relevance of polymicrobial versus monomicrobial bloodstream infections remains inconsistent. While some studies reported a significantly increased mortality in polymicrobial bloodstream infections [3, 4], others found that polymicrobial infections were not independently associated with worse outcomes [5, 6]. However, many existing studies suffer from significant limitations that challenge their generalizability, including small sample sizes, and restriction to selected subpopulations such as neutropenic or oncologic patients [3, 7, 8]. Additionally, there are only a few large-scale, ICU-focused analyses. If polymicrobial infections do not provide additional prognostic information, categorizing them as inherently more severe could lead to inaccurate risk assessments and less targeted care. To address these uncertainties, we conducted a large-scale retrospective cohort study in ICU patients with microbiologically confirmed bloodstream infections. By comparing outcomes in patients with monomicrobial versus polymicrobial infections, we aimed to better understand whether the presence of multiple pathogens truly translates into worse clinical outcomes in this vulnerable population.

## METHODS

### Study Design and Participants

This retrospective, inverse-probability weighted, covariate-adjusted cohort study included adult patients aged 18 years or older who were admitted to one of the 16 ICUs (surgical, medical, or emergency medicine units) of a tertiary-care hospital (University Hospital of Vienna, Medical University of Vienna, Austria) between March 2014 and March 2024. Eligible patients had an ICU length of stay of at least three days and at least one positive bacterial blood culture during their ICU admission. Patients were categorized into two groups based on the microbial composition of the index culture (ie, the first culture fulfilling the respective group criteria): patients with polymicrobial bloodstream infections, defined as the detection of two or more distinct bacterial pathogens in a single blood culture set (polymicrobial group) versus those with monomicrobial bloodstream infections, defined as the detection of exactly one bacterial pathogen (monomicrobial group). For patients with polymicrobial infections, the index culture was the first positive polymicrobial culture, regardless of whether earlier monomicrobial cultures had been identified. To address the potential misclassification due to contamination by skin flora, a secondary analysis was performed, in which common commensal organisms were excluded from both polymicrobial and monomicrobial classifications (common commensal-free cohort). The list of excluded organisms was based on the Centers for Disease Control and Prevention (CDC) guidelines for common blood culture contaminants and is provided in Supplementary Table 1 [9]. In the common commensal-free cohort, patients were reclassified into the monomicrobial or polymicrobial group based on the revised composition of the index blood culture. For example, if a polymicrobial culture was downgraded to monomicrobial due to the exclusion of a common commensal pathogen, the patient was reassigned to the monomicrobial cohort. Patients were excluded from the secondary analysis if no eligible culture remained after this reclassification. The commensal-free cohort was analyzed separately.

The research adhered to ethical principles in line with the Declaration of Helsinki. The project received approval from the local Ethics Committee (Ethics Committee of the Medical University of Vienna) with the reference EC 1348/2024.

### Outcomes

The primary outcome was all-cause mortality at 90 days of the index blood culture. Secondary outcomes included all-cause mortality at 30 days, microbiological failure between days 5 and 14, onset of fever occurring between days 2 and 14, development of acute respiratory distress syndrome (ARDS) within 7 days, initiation of extracorporeal membrane oxygenation (ECMO) within 7 days, and the duration of ICU and total hospital stay. The pathogen distribution was analyzed descriptively. Multidrug resistance (MDR) definitions follow the European Center for Disease Prevention and Control criteria [10].

Microbiological failure was further categorized into two separate secondary endpoints: pathogen-specific failure, defined as a positive blood culture between days 5 and 14 with at least one organism matching a pathogen identified in the index culture; and non-specific failure, defined as the detection of a different bacterial species during the same period. Fever was defined as any documented body temperature ≥38.0°C recorded between 48 h and 7 days after the index culture. ARDS was defined based on a modified Berlin definition, restricted to a PaO₂/FiO₂ ratio ≤300 mmHg occurring within 7 days of the index blood culture [11]. Due to limitations in data availability, radiographic criteria and exclusion of cardiogenic edema could not be assessed as part of the Berlin definition of ARDS.

### Data Sources and Variables

Clinical data were extracted from centrally stored electronic medical records of the hospital and included age, sex, comorbidities, the sequential organ failure assessment (SOFA) score, ICU type of primary ICU admission (medical, surgical, emergency), and treatments administered during ICU stay, including mechanical ventilation, vasopressors, corticosteroids, antibiotics, and ECMO. All time-related variables refer to the date that the index blood culture was collected (ie, the index date). Antimicrobial exposure prior to the index culture and relevant clinical parameters such as body temperature and PaO₂/FiO₂ ratios assessed immediately before or at the time of the positive index culture were defined as baseline variables.

### Statistical Analysis

Baseline characteristics were reported descriptively for both groups using mean ± standard deviation (SD), medians with interquartile range (IQR), or number (%). To address the risk of confounding, we used propensity score-based inverse probability weighting (IPW) and covariate-adjusted regression models to adjust for baseline differences between the polymicrobial and monomicrobial groups, as previously described [12]. Propensity scores were estimated using logistic regression, with the microbial group assignment (ie, monomicrobial or polymicrobial) serving as the dependent variable and all baseline covariates included as predictors. Covariates included age, sex, comorbidities, SOFA score, ICU type, timing of infection, prior antibiotic and corticosteroid use, and need for organ support. Variables were selected based on their potential interference with the group assignment (ie, polymicrobial or monomicrobial blood cultures) and clinical outcomes. The exact list of variables can be found in Supplementary Table 2. Stabilized weights were computed and trimmed at the 95th percentile to reduce the influence of extreme values. Covariate balance was assessed using standardized mean differences (SMDs), with values below 0.1 indicating acceptable balance [13]. Supplementary Figures 1 and 2 show the SMDs before and after covariate balancing in the overall and common commensal-free cohort. All SMDs were below 0.1 after IPW. Supplementary Figures 3 and 4 show the diagnostic plots and collinearity of covariates of the model used for the overall cohort, respectively. Supplementary Figures 5 and 6 show the diagnostic plots and collinearity of covariates of the model used for the common commensal-free cohort, respectively. Model diagnostics suggested no influential data points and acceptable covariate collinearity. Adjusted risk and mean differences with 95% confidence intervals were estimated using generalized linear models with robust standard errors, applied to the IPW-adjusted data. The same covariates used to generate the propensity scores were included in the outcome models to ensure robustness. All analyses were conducted separately for both the overall and commensal-free cohorts. Exploratory subgroup analyses were performed.

Furthermore, we performed an exploratory analysis within the polymicrobial cohort to compare outcomes between infections composed of all gram-negative organisms, all gram-positive organisms, or a mixture of gram-negative and gram-positive organisms. In a sensitivity analysis, common commensal pathogens detected in an index culture were considered clinically relevant if the same organism was subsequently isolated in a later culture, and patients were reclassified accordingly.

To further describe pathogen relationships in polymicrobial infections, co-occurrence networks were constructed based on the composition of polymicrobial index cultures. Each unique pair of co-isolated pathogens was treated as an edge in the network, with edge weights proportional to their frequency. The Fruchterman-Reingold force-directed layout was used for visualization, and community structure was explored using the Louvain clustering algorithm. Network construction and visualization were performed using the igraph and ggraph packages in R.

No formal sample size calculation was performed. Instead, a pragmatic approach was taken by including all eligible patients from the past 10 years, aiming to maximize sample size while minimizing potential confounding from long-term changes in clinical practice.

All statistical analyses were conducted using R version 4.1.2 (R Foundation for Statistical Computing, Vienna, Austria) and RStudio (RStudio Team, 2020). All reported *P*-values were two-sided, with values below 0.05 considered statistically significant. Given the exploratory nature of this study, no formal correction for multiple comparisons was applied. The results should be interpreted accordingly.

## RESULTS

### Participants

**Figure 1.**
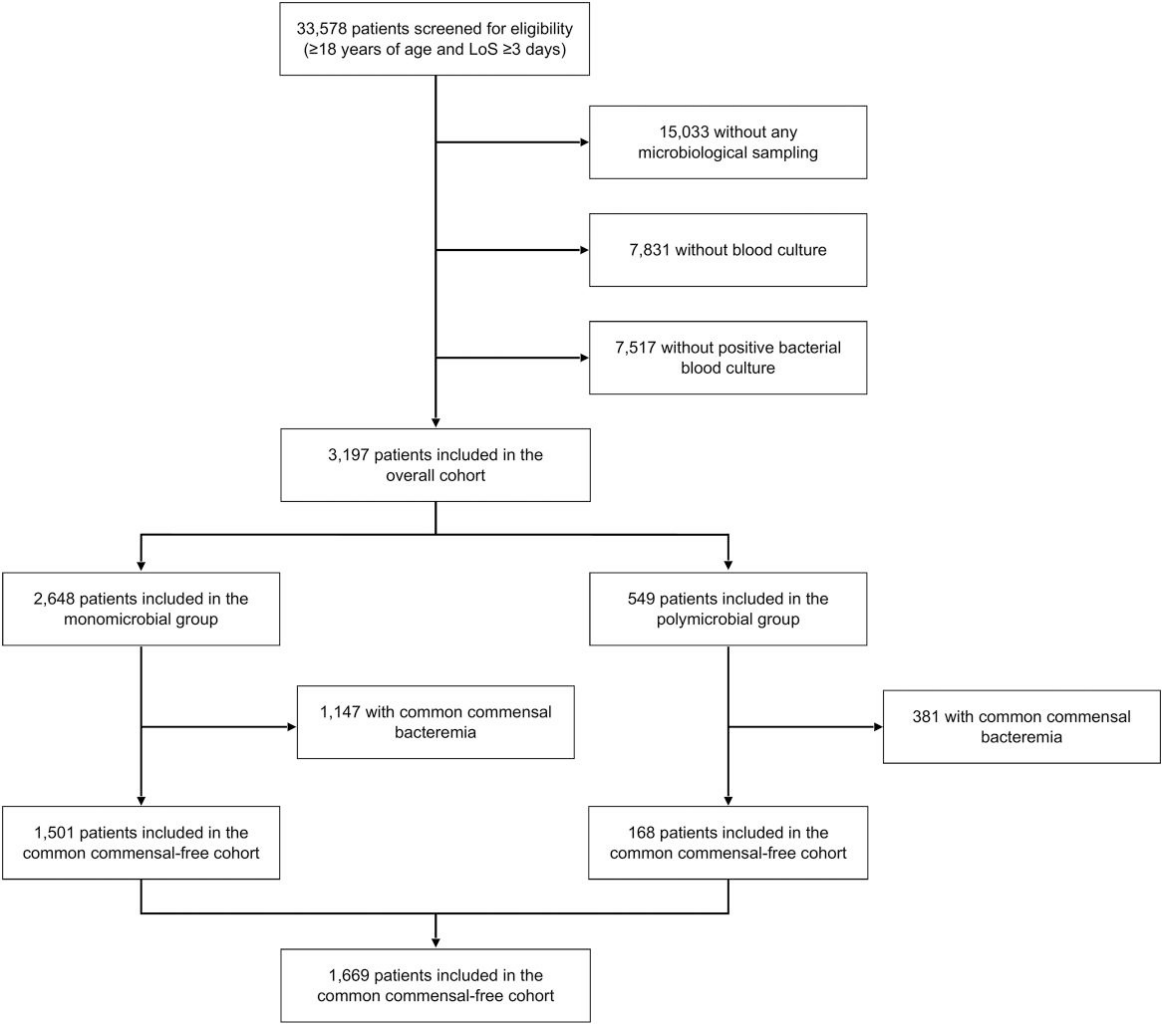
Flow chart of the study population.

Between March 2014 and March 2024, 33,578 patients ≥18 years of age were admitted to the ICU for at least 3 days and were screened for eligibility (Figure 1). After excluding patients without positive bacterial blood cultures, 3197 patients were included in the overall cohort analysis, of which 2648 were included in the monomicrobial group and 549 were included in the polymicrobial group. After excluding common commensal pathogens as defined by the CDC (Supplementary Table 1), 1669 patients remained in the common commensal-free cohort, including 1501 in the monomicrobial group and 168 in the polymicrobial group. Table 1 summarizes the patient characteristics of the overall study population.

**Table 1.** Baseline Characteristics of Participants in the Overall Cohort

*n*	Overall	Monomicrobial	Polymicrobial	*P*
	3197	2648	549	
Age, years (median [IQR])	61.0 [50.0–70.0]	61.0 [50.0–70.3]	59.0 [50.0–70.0]	.383
Age category (%)				.306
49 y or younger	586 (19.5)	493 (19.8)	93 (18.2)	
50–64 y	1150 (38.3)	937 (37.6)	213 (41.7)	
65–74 y	781 (26.0)	659 (26.4)	122 (23.9)	
74–79 y	286 (9.5)	242 (9.7)	44 (8.6)	
80 y or older	201 (6.7)	162 (6.5)	39 (7.6)	
Female sex (%)	1145 (35.8)	954 (36.0)	191 (34.8)	.616
Geographical origin (%)				.553
Africa	22 (0.7)	18 (0.7)	4 (0.7)	
Europe	2703 (84.5)	2249 (84.9)	454 (82.7)	
Middle East or Asia	50 (1.6)	40 (1.5)	10 (1.8)	
North or South America	13 (0.4)	12 (0.5)	1 (0.2)	
Days from ICU admission to index culture (median [IQR])	6.0 [1.0–14.0]	6.0 [1.0–13.0]	7.0 [1.0–17.0]	<.001
Days from ICU admission to index culture (category)				<.001
Day 1–2	1189 (37.2)	1017 (38.4)	172 (31.3)	
Day 3–7	614 (19.2)	506 (19.1)	108 (19.7)	
Day 8–14	628 (19.6)	530 (20.0)	98 (17.9)	
Day 15 or later	766 (24.0)	595 (22.5)	171 (31.1)	
Number of bacterial pathogens in index culture				<.001
1	2648 (82.8)	2648 (100.0)	0 (0.0)	
2	487 (15.2)	0 (0.0)	487 (88.7)	
3	52 (1.6)	0 (0.0)	52 (9.5)	
4	8 (0.3)	0 (0.0)	8 (1.5)	
5	1 (0.0)	0 (0.0)	1 (0.2)	
6	1 (0.0)	0 (0.0)	1 (0.2)	
Time period of admission (%)	…	…	…	.809
2014/15	508 (15.9)	418 (15.8)	90 (16.4)	
2016/17	645 (20.2)	531 (20.1)	114 (20.8)	
2018/19	410 (12.8)	337 (12.7)	73 (13.3)	
2020/21	772 (24.1)	640 (24.2)	132 (24.0)	
2022/23	781 (24.4)	658 (24.8)	123 (22.4)	
2024	81 (2.5)	64 (2.4)	17 (3.1)	
Type of ICU (%)				.158
Emergency Medicine ICU	198 (6.2)	160 (6.0)	38 (6.9)	
Medical ICU	1643 (51.4)	1345 (50.8)	298 (54.3)	
Surgical ICU	1356 (42.4)	1143 (43.2)	213 (38.8)	
Endocrine, nutritional, and metabolic diseases (%)	594 (18.6)	497 (18.8)	97 (17.7)	.587
Diabetes (%)	497 (15.5)	397 (15.0)	100 (18.2)	.067
Chronic kidney disease (%)	985 (30.8)	824 (31.1)	161 (29.3)	.437
Diseases of the digestive system (%)	582 (18.2)	478 (18.1)	104 (18.9)	.666
Hypertensive disease (%)	2549 (79.7)	2111 (79.7)	438 (79.8)	1.000
Ischemic heart disease (%)	1102 (34.5)	920 (34.7)	182 (33.2)	.506
Cerebrovascular disease (%)	245 (7.7)	191 (7.2)	54 (9.8)	.044
Arterial disease (%)	293 (9.2)	256 (9.7)	37 (6.7)	.037
Neoplasms (%)	418 (13.1)	342 (12.9)	76 (13.8)	.605
Diseases of the respiratory system (%)	728 (22.8)	604 (22.8)	124 (22.6)	.954
Diseases of the nervous system (%)	312 (9.8)	249 (9.4)	63 (11.5)	.159
Diseases of the skin and subcutaneous tissue (%)	104 (3.3)	84 (3.2)	20 (3.6)	.664
Mechanical ventilation at time of index culture (%)	781 (24.4)	661 (25.0)	120 (21.9)	.137
ECMO at index culture (%)	116 (3.6)	96 (3.6)	20 (3.6)	1.000
SOFA score (median [IQR])	7.0 [4.0, 10.0]	7.0 [4.0, 10.0]	7.0 [4.0, 10.0]	.337
SOFA Score (%)				.293
0–6	833 (26.1)	673 (25.4)	160 (29.1)	
7–9	457 (14.3)	377 (14.2)	80 (14.6)	
10 or higher	509 (15.9)	426 (16.1)	83 (15.1)	
Not available	1398 (43.7)	1172 (44.3)	226 (41.2)	
PaO_2_/FiO_2_ (%)				.998
100 mmHg or less	409 (12.8)	337 (12.7)	72 (13.1)	
100–199 mmHg	779 (24.4)	644 (24.3)	135 (24.6)	
200–299 mmHg	347 (10.9)	288 (10.9)	59 (10.7)	
300 mmHg or more	131 (4.1)	108 (4.1)	23 (4.2)	
Not available	1531 (47.9)	1271 (48.0)	260 (47.4)	
Fever (%)				.044
37.5°C or less	374 (11.7)	305 (11.5)	69 (12.6)	
37.6°C to 37.9°C	230 (7.2)	197 (7.4)	33 (6.0)	
38.0°C to 39.9°C	801 (25.1)	650 (24.5)	151 (27.5)	
40.0°C or more	47 (1.5)	33 (1.2)	14 (2.6)	
Not available	1745 (54.6)	1463 (55.2)	282 (51.4)	
Vasopressor at time of index culture (%)	1726 (54.0)	1431 (54.0)	295 (53.7)	.933
Inotrope at time of index culture (%)	388 (12.1)	333 (12.6)	55 (10.0)	.110
Antimicrobial treatment at time of index culture (%)	1827 (57.1)	1513 (57.1)	314 (57.2)	1.000
Corticosteroid treatment at time of index culture (%)	664 (20.8)	541 (20.4)	123 (22.4)	.327
Immunosuppression at time of index culture (%)	215 (6.7)	184 (6.9)	31 (5.6)	.310
Leukocytes at time of index culture, G/L (mean (SD))	12.60 (9.9)	12.60 (9.5)	12.60 (11.3)	.999
Leukocytes category, G/L (%)				.815
<4	244 (7.6)	202 (7.6)	42 (7.7)	
4–9	1155 (36.1)	953 (36.0)	202 (36.8)	
10–13	811 (25.4)	666 (25.2)	145 (26.4)	
14 or more	984 (30.8)	824 (31.1)	160 (29.1)	
Not available	3 (0.1)	3 (0.1)	0 (0.0)	
C-reactive protein at index culture, mg/dL (mean (SD))	14.33 (10.8)	14.49 (10.8)	13.54 (10.9)	.058
C-reactive protein category, mg/dL (%)				.124
<0.5	55 (1.7)	43 (1.6)	12 (2.2)	
0.5–9	1322 (41.4)	1072 (40.5)	250 (45.5)	
10–19	960 (30.0)	805 (30.4)	155 (28.2)	
20 or more	853 (26.7)	723 (27.3)	130 (23.7)	
Not available	7 (0.2)	5 (0.2)	2 (0.4)	
Procalcitonin at index culture, ng/mL (mean (SD))	10.27 (22.5)	10.14 (22.2)	10.85 (24.1)	.622
Procalcitonin category, ng/mL (%)				.062
<0.5	604 (18.9)	479 (18.1)	125 (22.8)	
0.5–1.9	382 (11.9)	313 (11.8)	69 (12.6)	
02–9.9	298 (9.3)	251 (9.5)	47 (8.6)	
10 or higher	331 (10.4)	270 (10.2)	61 (11.1)	
Not available	1582 (49.5)	1335 (50.4)	247 (45.0)	
Creatinine at index culture, mg/dL (mean (SD))	1.81 (1.9)	1.84 (1.9)	1.65 (1.8)	.034
Creatinine protein category, mg/dL (%)	…	…	…	.139
<0.9	1424 (44.5)	1153 (43.5)	271 (49.4)	
1.0–1.4	586 (18.3)	486 (18.4)	100 (18.2)	
01.5–1.9	315 (9.9)	264 (10.0)	51 (9.3)	
2.0–2.9	355 (11.1)	303 (11.4)	52 (9.5)	
3.0 or higher	503 (15.7)	431 (16.3)	72 (13.1)	
Not available	14 (0.4)	11 (0.4)	3 (0.5)	
Direct ICU admission (%)	1626 (50.9)	1339 (50.6)	287 (52.3)	.495
Community-acquired infection (%)	776 (24.3)	670 (25.3)	106 (19.3)	.003

In the overall cohort, baseline characteristics were generally well-balanced between monomicrobial and polymicrobial groups. The median age was comparable (61 vs 59 years), and the proportion of female patients did not differ significantly (36.0% in the monomicrobial vs 34.8% in the polymicrobial group). Comorbidity prevalence was similar between the groups. The most frequent comorbidities in both groups were hypertensive disease (79.7% vs 79.8%), ischemic heart disease (34.7% vs 33.2%), and chronic kidney disease (31.1% vs 29.3%).

While the median day of the index culture was day 6 in the monomicrobial and day 7 in the polymicrobial group, the distribution differed significantly, with a higher proportion of polymicrobial infections occurring on or after day 15 (22.5% vs 31.1%).

ICU admission type (ie, primary admission to an emergency medicine-, medical-, or surgical ICU) and illness severity were evenly distributed across groups. SOFA scores at the time of index culture were balanced (median 7 [IQR 4–10] in both groups), as were the rates of organ support, including mechanical ventilation, vasopressor use, inotrope use, and ECMO (Table 1). Fever at baseline was more common in the polymicrobial group (30.1%) than in the monomicrobial group (25.7%). Finally, baseline medical treatment with antibiotics, corticosteroids, and immunosuppressants was similar between the groups.

Similarly, in the common commensal-free cohort, baseline characteristics were well-balanced (Supplementary Table 3).

### Primary Endpoint

**Figure 2.**
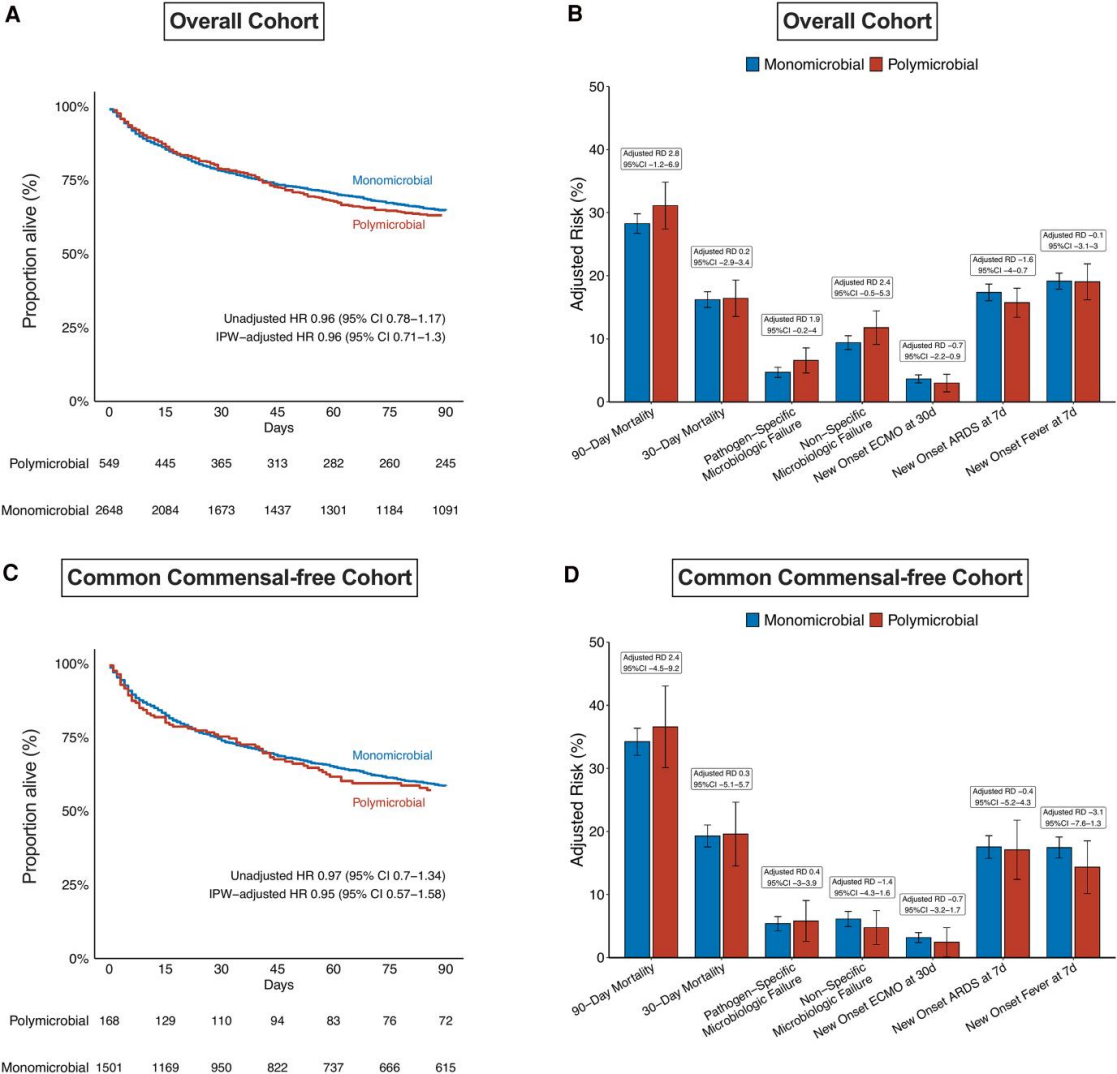
Primary and secondary outcomes in the (*A*, *B*) overall and (*C*, *D*) common commensal-free cohorts.

In the overall cohort, 90-day all-cause mortality was similar between the monomicrobial and polymicrobial groups. At 90 days, death occurred in 749 of 2648 (28.3%, adjusted risk 28.3%) patients in the monomicrobial group and in 164 of 549 (29.9%, adjusted risk 31.1%) patients in the polymicrobial group, resulting in an adjusted risk difference (RD) of 2.84% points (95% CI, −1.19–6.88) (Table 2 and Figure 2). Similarly, in the common commensal-free group, 90-day all-cause death occurred in 512 of 1501 (34.1%, adjusted risk 34.2%) patients in the monomicrobial group and 61 of 168 (36.3%, adjusted risk 36.6%) patients in the polymicrobial group, resulting in an adjusted RD of 2.36% points (95% CI, −4.48–9.20) (Supplementary Table 4 and Figure 2).

**Table 2.** Primary and Secondary Outcomes in the Overall Cohort

Group	*N*	Unadjusted Risk or Mean (95% CI)	Unadjusted RD or mean difference (95% CI)	Unadj. *P*	Adjusted Risk or Mean (95% CI)	Adjusted RD or mean difference (95% CI)	Adj. *P*
Total sample size							
Monomicrobial	2648						
Polymicrobial	549						
Death at 90 d							
Monomicrobial	749	28.3% (26.5%–30.0%)			28.3% (26.7%–29.8%)		
Polymicrobial	164	29.9% (26.0%–33.7%)	1.59% (−2.61%–5.78%)	0.46	31.11% (27.4%–34.8%)	2.84% (−1.19%–6.88%)	0.17
Death at 30 d							
Monomicrobial	431	16.3% (14.9%–17.7%)			16.2% (15.0%–17.4%)		
Polymicrobial	87	15.9% (12.8%–18.9%)	−0.43% (−3.79%–2.93%)	0.8	16.4% (13.5%–19.3%)	0.21% (−2.93%–3.36%)	0.9
Microbiological failure (any pathogen)							
Monomicrobial	249	9.4% (8.29%–10.5%)			9.38% (8.29%–10.5%)		
Polymicrobial	70	12.8% (9.96%–15.5%)	3.35% (0.34%–6.35%)	0.029	11.8% (9.11%–14.4%)	2.38% (−0.5%–5.26%)	0.11
Microbiological failure (same pathogen)							
Monomicrobial	125	4.72% (3.91%–5.53%)			4.67% (3.88%–5.47%)		
Polymicrobial	40	7.29% (5.11%–9.46%)	2.57% (0.25%–4.88%)	0.03	6.56% (4.58%–8.54%)	1.89% (−0.25%–4.02%)	0.083
New onset ECMO at 30 d							
Monomicrobial	97	3.66% (2.95%–4.38%)			3.65% (3.02%–4.29%)		
Polymicrobial	16	2.91% (1.51%–4.32%)	−0.75% (−2.33%–0.83%)	0.35	2.99% (1.58%–4.39%)	−0.67% (−2.21%–0.88%)	0.4
New onset ARDS at 7 d							
Monomicrobial	457	17.3% (15.8%–18.7%)			17.4% (16.0%–18.7%)		
Polymicrobial	92	16.8% (13.6%–19.9%)	−0.5% (−3.94%–2.94%)	0.78	15.7% (13.4%–18.0%)	−1.65% (−3.99%–0.7%)	0.17
Fever at 7 d							
Monomicrobial	494	18.7% (17.2%–20.1%)			19.1% (17.9%–20.4%)		
Polymicrobial	115	21.0% (17.5%–24.4%)	2.29% (−1.42%–6%)	0.23	19.0% (16.2%–21.9%)	−0.1% (−3.15%–2.95%)	0.95
Length of ICU stay (days after index culture)							
Monomicrobial		19.6 (18.7–20.4)			19.6 (18.7–20.4)		
Polymicrobial		20.1 (18.2–22.0)	0.54 (−1.56–2.64)	0.61	19.8 (17.9–21.7)	0.23 (−1.82–2.28)	0.83
Length of hospital stay (days after index culture)							
Monomicrobial		42.4 (39.8–45.0)			42.6 (40.6–44.6)		
Polymicrobial		48.3 (42.6–54.0)	5.85 (−0.42–12.1)	0.067	45.2 (37.2–53.2)	2.62 (−5.65–10.9)	0.54

### Secondary Endpoints

#### 30-day All-cause Mortality

The 30-day all-cause mortality was comparable between the monomicrobial and polymicrobial groups in the overall cohort (adjusted RD, 0.21%; 95% CI, −2.93–3.36) and common-commensal-free cohort (adjusted RD, 0.31; 95% CI, −5.06–5.67) (Table 2 and Supplementary Table 4).

#### Microbiological Failure

Pathogen-specific microbiological failure occurred in 125 of 2648 (4.72%, adjusted risk 4.67%) patients in the monomicrobial group and 40 of 549 (7.29%, adjusted risk 6.56%) patients in the polymicrobial group, yielding an adjusted RD of 1.89% (95% CI, −0.25% to 4.02%) (Table 2 and Figure 2). In the common commensal-free cohort, pathogen-specific microbiological failure occurred in 81 of 1501 (5.40%, adjusted risk 5.38%) patients in the monomicrobial group and 11 of 168 (6.55%, adjusted risk 5.85%) patients in the polymicrobial group (adjusted RD, 0.42%; 95% CI, −3.02% to 3.87%).

In the overall cohort, non-specific microbiological failure occurred in 249 of 2648 (9.40%, adjusted risk 9.38%) patients in the monomicrobial group and 70 of 549 (12.75%, adjusted risk 11.76%) patients in the polymicrobial group, resulting in an adjusted RD of 2.38% (95% CI, −0.50% to 5.26%) (Table 2 and Figure 2). In the common commensal-free cohort, non-specific microbiological failure occurred in 92 of 1501 (6.13%, adjusted risk 6.12%) patients in the monomicrobial group and 11 of 168 (6.55%, adjusted risk 4.76) patients in the polymicrobial group (adjusted RD, −1.36%; 95% CI, −4.31% to 1.59%) (Supplementary Table 4 and Figure 2).

#### Other Secondary Outcomes

The remaining secondary outcomes of the overall cohort are summarized in Table 2 and Figure 2. The remaining secondary outcomes of the common commensal-free cohort are summarized in Supplementary Table 4 and Figure 2. New initiation of ECMO at 30 days, new onset of ARDS at 7 days, and new onset of fever at 7 days were similar between the monomicrobial and polymicrobial groups in the overall cohort and in the common-commensal-free cohort. Length of ICU stay and length of hospital stay were similar between the monomicrobial and polymicrobial groups in the overall cohort and the common-commensal-free cohort.

### Pathogen Distribution

**Figure 3.**
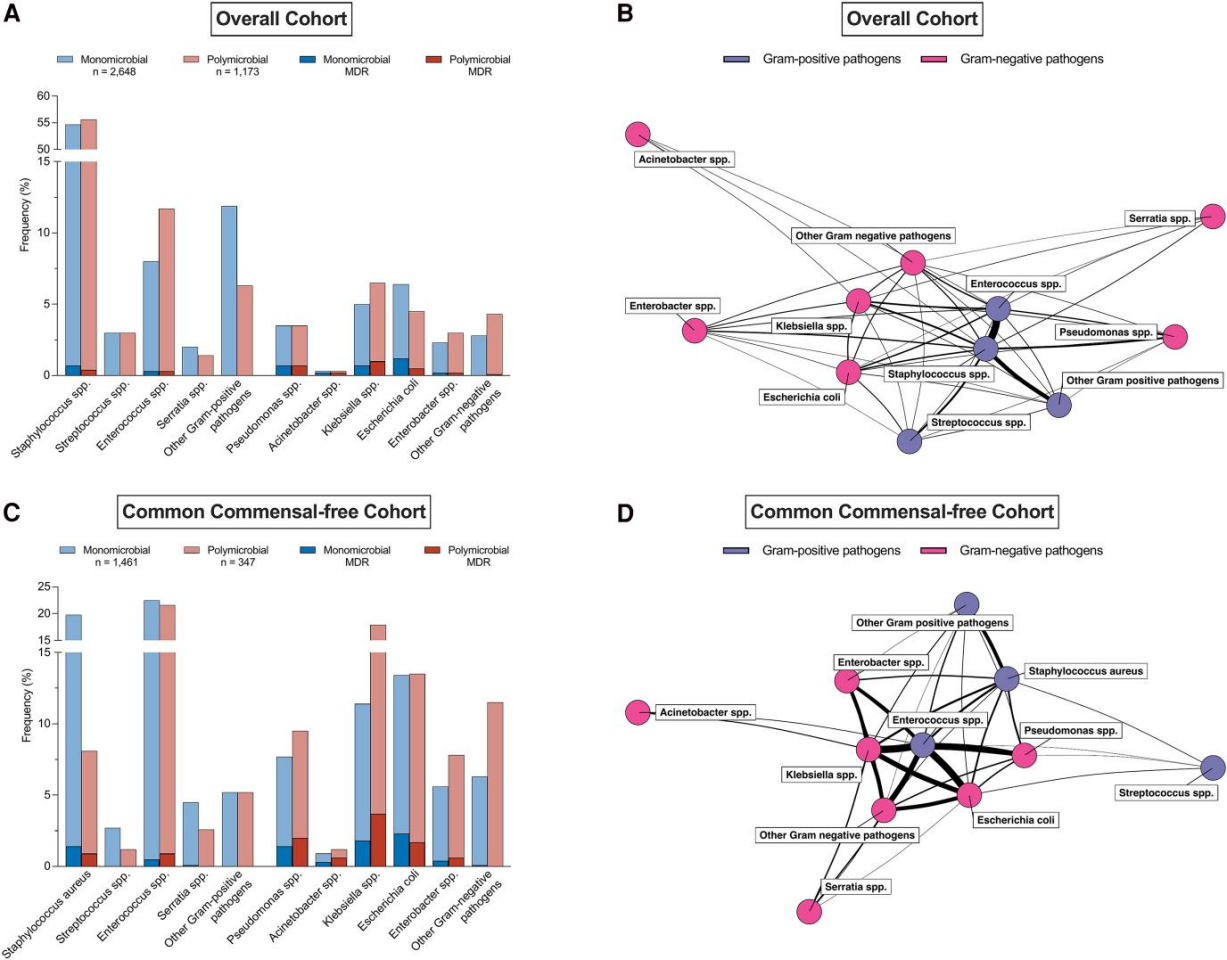
Prevalence (*A* and C) and co-occurrence (*B* and *D*) of bacterial pathogens. Relative frequencies (%) of bacterial pathogens and prevalence of Multidrug-Resistant (MDR) pathogens in blood cultures in (*A*) the overall and, (*C*) common commensal-free cohort; Shaded areas represent the proportion of MDR strains of the respective pathogens. Network plots of co-occurring pathogens in (*B*) the overall and (*D*) common commensal-free cohort; Node color indicates Gram category; edge thickness reflects the frequency of co-occurrence; node proximity is determined by overall network connectivity.

Figure 3 and Table 3 depict and summarize the distribution of pathogens and the proportion of MDR organisms identified in bloodstream infections in the overall and common commensal-free cohort.

**Table 3.** Prevalence of Bacterial Pathogens in Overall and Common Commensal-free Cohorts

Pathogen	Overall *N* (%)		Monomicrobial *N* (%)		Polymicrobial *N* (%)	
	Total	MDR	Total	MDR	Total	MDR
Overall cohort						
Gram-positive pathogens						
*Staphylococcus* spp.	2100 (55.0)	24 (0.6)	1448 (54.7)	19 (0.7)	652 (55.6)	5 (0.4)
*Streptococcus* spp.	114 (3.0)	0 (0.0)	79 (3.0)	0 (0.0)	35 (3.0)	0 (0.0)
*Enterococcus* spp.	350 (9.2)	10 (0.3)	213 (8.0)	7 (0.3)	137 (11.7)	3 (0.3)
*Serratia* spp.	68 (1.8)	1 (0.0)	52 (2.0)	1 (0.0)	16 (1.4)	0 (0.0)
Other Gram-positive pathogens	389 (10.2)	0 (0.0)	315 (11.9)	0 (0.0)	74 (6.3)	0 (0.0)
Gram-negative pathogens						
*Pseudomonas* spp.	135 (3.5)	26 (0.7)	94 (3.5)	18 (0.7)	41 (3.5)	8 (0.7)
*Acinetobacter* spp.	13 (0.3)	6 (0.2)	9 (0.3)	4 (0.2)	4 (0.3)	2 (0.2)
*Klebsiella* spp.	208 (5.4)	31 (0.8)	132 (5.0)	19 (0.7)	76 (6.5)	12 (1.0)
*Escherichia coli*	223 (5.8)	37 (1.0)	170 (6.4)	31 (1.2)	53 (4.5)	6 (0.5)
*Enterobacter* spp.	96 (2.5)	7 (0.2)	61 (2.3)	5 (0.2)	35 (3.0)	2 (0.2)
Other Gram-negative pathogens	125 (3.3)	1 (0.0)	75 (2.8)	0 (0.0)	50 (4.3)	1 (0.1)
Common commensal-free cohort						
Gram-positive pathogens						
*Staphylococcus aureus*	317 (17.5)	24 (1.3)	289 (19.8)	21 (1.4)	28 (8.1)	3 (0.9)
*Streptococcus* spp.	43 (2.4)	0 (0.0)	39 (2.7)	0 (0.0)	4 (1.2)	0 (0.0)
*Enterococcus* spp.	404 (22.3)	10 (0.6)	329 (22.5)	7 (0.5)	75 (21.6)	3 (0.9)
*Serratia* spp.	75 (4.1)	2 (0.1)	66 (4.5)	2 (0.1)	9 (2.6)	0 (0.0)
Other Gram-positive pathogens	94 (5.2)	0 (0.0)	76 (5.2)	0 (0.0)	18 (5.2)	0 (0.0)
Gram-negative pathogens						
*Pseudomonas* spp.	145 (8.0)	27 (1.5)	112 (7.7)	20 (1.4)	33 (9.5)	7 (2.0)
*Acinetobacter* spp.	17 (0.9)	7 (0.4)	13 (0.9)	5 (0.3)	4 (1.2)	2 (0.6)
*Klebsiella* spp.	229 (12.7)	39 (2.2)	167 (11.4)	26 (1.8)	62 (17.9)	13 (3.7)
*Escherichia coli*	243 (13.4)	39 (2.2)	196 (13.4)	33 (2.3)	47 (13.5)	6 (1.7)
*Enterobacter* spp.	109 (6.0)	8 (0.4)	82 (5.6)	6 (0.4)	27 (7.8)	2 (0.6)
Other Gram-negative pathogens	132 (7.3)	1 (0.1)	92 (6.3)	1 (0.1)	40 (11.5)	0 (0.0)

Data is presented as *n* (%).

In the overall cohort, a total number of 3812 pathogens were isolated, of which 3012 (79.0%) were Gram-positive and 800 (21.0%) were Gram-negative. The most frequent pathogens in the monomicrobial group were *Staphylococcus* spp. (1448 [54.7%] of 2648), *Enterococcus* spp. (213 [8.0%] of 2648), and *Escherichia coli* (170 [6.4%] of 2648). In the polymicrobial group, *Staphylococcus* spp. (652 [55.6%] of 1173), *Enterococcus* spp. (137 [11.7%] of 1173), and *Klebsiella* spp. (76 [6.5%] of 1173) were the most common. The highest percentages of MDR isolates were observed in *Acinetobacter* spp. (6 [46.2%] of 13), *Pseudomonas* spp. (26 [19.3%] of 135), and *Escherichia coli* (37 [16.6%] of 223).

In the common commensal-free cohort, 1808 pathogens were identified, of which 933 (51.6%) were Gram-positive and 875 (48.4%) were Gram-negative. In the common commensal-free cohort, the most frequent pathogens in monomicrobial infections were *Staphylococcus aureus* (289 [19.8%] of 1461), *Enterococcus* spp. (329 [22.5%] of 1461), and *Escherichia coli* (196 [13.4%] of 1461). In polymicrobial infections, *Enterococcus* spp. (75 [21.6%] of 347), *Klebsiella* spp. (62 [17.9%] of 347), and *Escherichia coli* (47 [13.5%] of 347) were the most common.

### Antimicrobial Use

Supplementary Table 5 summarizes the antimicrobial treatment administered after the collection of the index culture in the overall cohort. Antimicrobial therapy was similar between groups (86.4% in the monomicrobial group vs 88.9% in the polymicrobial group). The use of broad-spectrum agents such as piperacillin-tazobactam (38.4% vs 42.3%) and carbapenems (32.5% vs 33.9%) was frequent in both groups. In the common commensal-free cohort (Supplementary Table 6), antimicrobial use was similarly high (85.7% in the monomicrobial group vs 88.7% in polymicrobial infections). Polymicrobial infections were more frequently treated with piperacillin-tazobactam (50.0% vs 39.9%, *P* = .015), carbapenems (43.5% vs 35.3%, *P* = .046), and fosfomycin (15.5% vs 9.0%, *P* = .01).

### Exploratory Analyses

**Figure 4.**
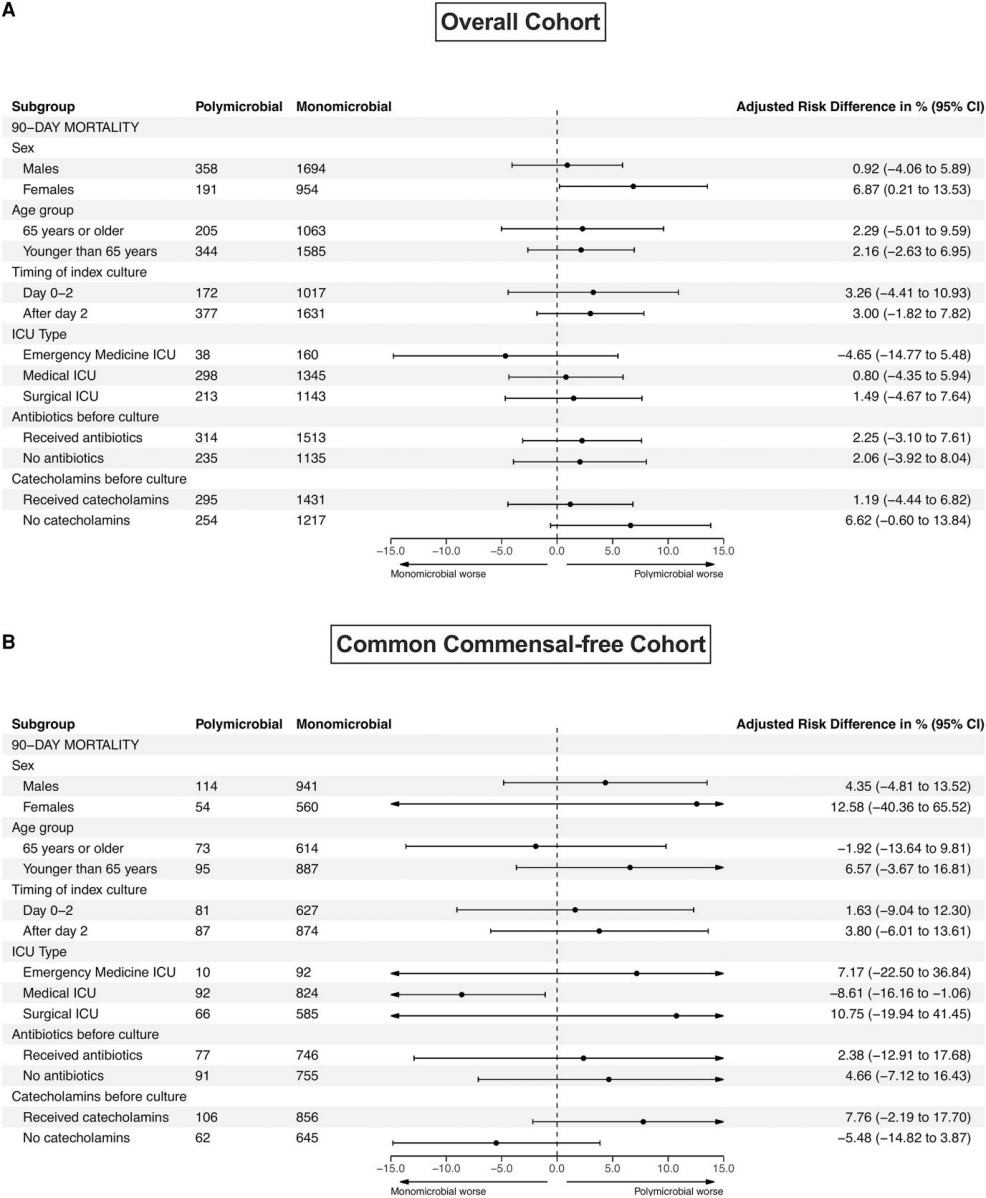
Subgroup analysis in (*A*) overall and (*B*) common commensal-free cohort.

Subgroup analyses of 90-day all-cause mortality are presented in Figure 4. In the overall cohort, the adjusted risk difference between polymicrobial and monomicrobial bloodstream infections was largely consistent across subgroups. However, polymicrobial infections were associated with a significantly higher risk of 90-day all-cause death in the subgroup of female patients (adjusted RD 6.87%, 95% CI 0.21–13.50). In the common commensal-free cohort, most subgroup effects were not statistically significant, with the exception of patients treated in a medical ICU, where polymicrobial infections were associated with a significantly lower adjusted mortality risk compared with monomicrobial infections (adjusted RD −8.61%, 95% CI −16.20 to −1.06). Results of the exploratory analysis of outcomes by type of polymicrobial infection are presented in Supplementary Figure 6. There were no significant differences in outcomes by type of polymicrobial infection, except for the 90-day all-cause mortality, which was 26.1% in the gram-positive polymicrobial group and 36.2% in the gram-negative polymicrobial group (unadjusted RD −10.1, 95% CI −19.2 to −0.01, *P* = .03).

In the sensitivity analysis, where repeated common commensal isolates were considered clinically relevant, outcomes remained similar to both the overall and common-commensal-free cohorts (Supplementary Table 7).

## DISCUSSION

In this large retrospective cohort of 3197 critically ill patients with bloodstream infections, polymicrobial and monomicrobial infections were associated with similar clinical and microbiological outcomes. Adjusted analyses revealed no significant difference in 90-day all-cause mortality or secondary endpoints. Although minor numerical trends suggested higher rates of pathogen-specific microbiological failure in polymicrobial infections, these differences were small, did not reach statistical significance, and did not translate into differences in more meaningful clinical outcomes. Importantly, these findings were consistent in a sensitivity analysis that excluded potential blood culture contaminants. By removing episodes involving common skin commensal pathogens, we minimized the risk of misclassifying contamination as true infection. The consistency of outcomes in the common commensal-free cohort reinforces the conclusion that the comparable mortality and other endpoints reflect the true clinical similarity between monomicrobial and polymicrobial bloodstream infections in critically ill patients. Subgroup analyses indicated a potential association between polymicrobial bloodstream infections and increased mortality among female patients in the overall cohort, whereas monomicrobial infections appeared to be linked with higher mortality in patients admitted to medical ICUs. However, given the number of comparisons performed and the lack of consistency of these findings across both cohorts, these results should be interpreted with caution and not considered conclusive.

Polymicrobial bloodstream infections are commonly believed to be particularly severe [14], partly because of the risk of inadequate empiric antibiotic coverage and their association with complex infection sources [15]. Emerging evidence suggests that the mortality difference between polymicrobial and monomicrobial bloodstream infections may have diminished over time [5], potentially as a result of advances in early administration of broad-spectrum antimicrobial therapy. Accordingly, in our study, patients in the polymicrobial group received more broad-spectrum beta-lactam antibiotics (mainly piperacillin-tazobactam and carbapenems) than patients with monomicrobial infections, likely reflecting empiric therapy prior to culture results. While this may improve outcomes, it also highlights antimicrobial stewardship considerations, as unnecessary broad-spectrum use can promote resistance.

Several analyzes agree with our findings that polymicrobial bloodstream infections are not associated with increased mortality. An observational study from Spain, including 75 polymicrobial bloodstream infections, found that these infections did not independently affect mortality after adjusting for confounders [16]. Similarly, a recent sepsis cohort study in South Korea, including 429 polymicrobial bloodstream infections, reported a crude hospital mortality increase for polymicrobial infections (36% vs 30%), but this difference disappeared after adjustment (adjusted HR 1.15, 95% CI 0.97–1.36) [5]. Conversely, some cohorts have reported significantly worse outcomes associated with polymicrobial bacteremia. A prospective study of 133 polymicrobial infections in Taiwan found that polymicrobial bloodstream infections were independently associated with higher 90-day mortality (adjusted OR 2.20, 95% CI 1.98–2.60) [17]. The discrepancies in the literature likely reflect differences in patient populations and the extent of confounder control. Polymicrobial infections may occur in patients with greater illness severity or in clinical contexts associated with inherently high mortality. Without proper adjustment for these underlying factors, polymicrobial infections may falsely appear to be independent predictors of poor outcomes. Unlike most previous studies [5, 17], the polymicrobial and monomicrobial groups had similar baseline characteristics and received comparable antimicrobial treatment, explaining why unadjusted and adjusted analyses of our study yielded similar results. Overall, our findings add to the growing body of evidence suggesting that the presence of multiple bloodstream pathogens is not an independent driver of mortality in the ICU. Consequently, in contemporary ICU settings with established treatment protocols and timely empiric broad-spectrum therapy, clinicians should not automatically anticipate poorer outcomes solely based on the detection of multiple pathogens.

### Strengths and Limitations

To our knowledge, this study examined the largest number of polymicrobial bloodstream infections in intensive care patients. We conducted detailed adjusted analyses, including IPW and a covariate-adjusted generalized model, which confirmed the unadjusted findings. The use of two separate analyses (overall and common commensal-free analysis) further strengthens our conclusions by addressing a key source of bias that may either involve the erroneous exclusion of opportunistic pathogens, which may be harmful to vulnerable patients, or the erroneous inclusion of contaminants.

Despite these strengths, the findings of this study should be interpreted in the context of its limitations. First, the retrospective observational design is inherently subject to residual confounding and bias. We relied on medical record data, and unmeasured factors, such as source control procedures or host immune status (not covered by comorbidities and use of immunosuppressants) could have influenced outcomes. Second, although no substantial differences were found in primary and secondary outcomes, it may have been underpowered to detect subtle differences or differences in rare complications. Third, we were not able to stratify results by infection source or pathogen type. Given that infection sources may independently influence clinical outcomes, the inability to stratify results by source limits the ability to draw nuanced conclusions. Notably, correctly identifying the source of bacteremia remains a challenge not only in retrospective studies but also in clinical practice, due to overlapping infection sites, non-specific clinical findings, and the complexity of critically ill patients. Fourth, a detailed assessment of whether patients received regimens active against all isolated organisms or the time to receipt of active antibiotics was not feasible. Even when culture results and antibiograms were available, susceptibility was not routinely tested for all potential antimicrobial agents, meaning such analyses would have relied on assumptions and could not be performed objectively. Fifth, although baseline characteristics between groups were similar, the median days from ICU admission to index culture were one day later in the polymicrobial group. Nevertheless, we included the time to index culture as a covariate in the adjusted analysis. Finally, our study was conducted in tertiary-care ICUs with established sepsis protocols, which may limit generalizability to other settings. Therefore, our findings should be applied cautiously across different healthcare contexts.

## CONCLUSIONS

In this retrospective cohort of critically ill patients with bacterial bloodstream infections, we found no significant differences in all-cause 90-day mortality, microbiological failure, or other clinical outcomes between monomicrobial and polymicrobial infections, before and after statistical adjustment for multiple covariates. These findings were consistent after the exclusion of common commensal organisms.

## Supplementary Material

ofaf591_Supplementary_Data

## Supplementary Data

Supplementary materials are available at *Open Forum Infectious Diseases* online. Consisting of data provided by the authors to benefit the reader, the posted materials are not copyedited and are the sole responsibility of the authors, so questions or comments should be addressed to the corresponding author.
