# Supplementary material for: Clinical and Microbiological Outcomes of Critically Ill Patients With Monomicrobial Versus Polymicrobial Bacterial Bloodstream Infections: A Retrospective Cohort Study
Source: Open Forum Infect Dis. 2025 Sep 24;12(10):ofaf591. doi: 10.1093/ofid/ofaf591 (PMC12502659; doi:10.1093/ofid/ofaf591)
Supplement: ofaf591_Supplementary_Data [file ofaf591_supplementary_data.docx]

# **SUPPLEMENTARY APPENDIX to:**

# **Clinical and microbiological outcomes of critically ill patients with monomicrobial versus polymicrobial bacterial bloodstream infections: a retrospective cohort study**

Anselm Jorda^1,†^,Felix Bergmann^1,†^, Marlene Prager^1^, Katarina Kumpf^2^, Georg Gelbenegger^1^, Markus Zeitlinger^1,*^

† Contributed equally

^1^Department of Clinical Pharmacology, Medical University of Vienna, Vienna, Austria

^2^IT Systems and Communications, Medical University of Vienna, Vienna, Austria

## ***Corresponding Author**

Markus Zeitlinger

Department of Clinical Pharmacology, Medical University of Vienna

Waehringer Guertel 18-20, 1090 Vienna, Austria

markus.zeitlinger@meduniwien.ac.at

Tel: +43 1 40400 29810

# **Supplementary Tables**

**Supplementary Table 1** Centers for Disease Control and Prevention (CDC) list of common skin commensal pathogens excluded in the common commensal-free analysis of this study

| **NHSN Code** | **NHSN Display Name** | **SNOMED Preferred Term** | **SNOMED Code** |
| --- | --- | --- | --- |
| ACTSP | Actinomyces | Actinomyces | 40560008 |
| ACTBO | Actinomyces bovis | Actinomyces bovis | 59806008 |
| ACTDENT | Actinomyces dentalis | Actinomyces dentalis | 426330001 |
| ACTFUNK | Actinomyces funkei | Actinomyces funkei | 419012004 |
| ACTGR | Actinomyces gerencseriae | Actinomyces gerencseriae | 113416002 |
| ACTGRAE | Actinomyces graevenitzii | Actinomyces graevenitzii | 113417006 |
| ACTIS | Actinomyces israelii | Actinomyces israelii | 46369004 |
| ACTNA | Actinomyces naeslundii | Actinomyces naeslundii | 8940004 |
| ACTORIC | Actinomyces oricola | Actinomyces oricola | 425488009 |
| ACTORIS | Actinomyces oris | Actinomyces oris | 447175005 |
| ACTRADI | Actinomyces radicidentis | Actinomyces radicidentis | 427691003 |
| ACTUROG | Actinomyces urogenitalis | Actinomyces urogenitalis | 409827009 |
| ACTVI | Actinomyces viscosus | Actinomyces viscosus | 33529006 |
| AEGU | Aerococcus | Aerococcus | 9008009 |
| AECH | Aerococcus christensenii | Aerococcus christensenii | 409818008 |
| AESGN | Aerococcus sanguinicola | Aerococcus sanguinicola | 427222006 |
| AEUR | Aerococcus urinae | Aerococcus urinae | 243230001 |
| AEURQ | Aerococcus urinaeequi | Aerococcus urinaeequi | 430979003 |
| AEURH | Aerococcus urinaehominis | Aerococcus urinaehominis | 409819000 |
| AEVI | Aerococcus viridans | Aerococcus viridans | 78803006 |
| ASNSP | Alpha-hemolytic Streptococcus, not S pneumoniae | Alpha-hemolytic Streptococcus not Streptococcus pneumoniae | 713921004 |
| ARCSP | Arcanobacterium | Arcanobacterium | 51714009 |
| ARCHA | Arcanobacterium haemolyticum | Arcanobacterium haemolyticum | 44723000 |
| ARCPLUR | Arcanobacterium pluranimalium | Arcanobacterium pluranimalium | 428939003 |
| ARTSP | Arthrobacter | Arthrobacter | 56214009 |
| ARTAGIL | Arthrobacter agilis | Arthrobacter agilis | 113432004 |
| ARTASTR | Arthrobacter astrocyaneus | Arthrobacter astrocyaneus | 113433009 |
| ARTCITR | Arthrobacter citreus | Arthrobacter citreus | 44955005 |
| ARTCRYS | Arthrobacter crystallopoietes | Arthrobacter crystallopoietes | 113435002 |
| ARTFLAV | Arthrobacter flavus | Arthrobacter flavus | 429762004 |
| ARTGAND | Arthrobacter gandavensis | Arthrobacter gandavensis | 428332000 |
| ARTGLOB | Arthrobacter globiformis | Arthrobacter globiformis | 3840003 |
| ARTKORE | Arthrobacter koreensis | Arthrobacter koreensis | 427847001 |
| ARTLUTE | Arthrobacter luteolus | Arthrobacter luteolus | 127497007 |
| ARTMETH | Arthrobacter methylotrophus | Arthrobacter methylotrophus | 428270003 |
| ARTNASI | Arthrobacter nasiphocae | Arthrobacter nasiphocae | 428337006 |
| ARTORYZ | Arthrobacter oryzae | Arthrobacter oryzae | 446485009 |
| ARTPASC | Arthrobacter pascens | Arthrobacter pascens | 113442002 |
| ARTPSYC | Arthrobacter psychrolactophilus | Arthrobacter psychrolactophilus | 428338001 |
| ARTRAMO | Arthrobacter ramosus | Arthrobacter ramosus | 113444001 |
| ARTRHOM | Arthrobacter rhombi | Arthrobacter rhombi | 428277000 |
| ARTROSE | Arthrobacter roseus | Arthrobacter roseus | 429003002 |
| ARTRUSS | Arthrobacter russicus | Arthrobacter russicus | 428049002 |
| ARTVISC | Arthrobacter viscosus | Arthrobacter viscosus | 113447008 |
| ARTWOLU | Arthrobacter woluwensis | Arthrobacter woluwensis | 113448003 |
| BAC | Bacillus | Bacillus | 44762009 |
| BAEOL | Bacillus aeolius | Bacillus aeolius | 428272006 |
| BAERI | Bacillus aerius | Bacillus aerius | 446486005 |
| BALCA | Bacillus alcalophilus | Bacillus alcalophilus | 90547001 |
| BALGI | Bacillus algicola | Bacillus algicola | 428278005 |
| BAMYL | Bacillus amyloliquefaciens | Bacillus amyloliquefaciens | 82289003 |
| BAQUI | Bacillus aquimaris | Bacillus aquimaris | 423188000 |
| BARSE | Bacillus arseniciselenatis | Bacillus arseniciselenatis | 429004008 |
| BASAH | Bacillus asahii | Bacillus asahii | 429115001 |
| BATRO | Bacillus atrophaeus | Bacillus atrophaeus | 113450006 |
| BAZOT | Bacillus azotoformans | Bacillus azotoformans | 72997009 |
| BBADI | Bacillus badius | Bacillus badius | 33457001 |
| BBATA | Bacillus bataviensis | Bacillus bataviensis | 429246006 |
| BBENZ | Bacillus benzoevorans | Bacillus benzoevorans | 39232002 |
| BBUTA | Bacillus butanolivorans | Bacillus butanolivorans | 447039005 |
| BCARB | Bacillus carboniphilus | Bacillus carboniphilus | 113451005 |
| BC | Bacillus cereus | Bacillus cereus | 84408007 |
| BCERG | Bacillus cereus group | Bacillus cereus group | 413618005 |
| BCHIT | Bacillus chittonlyticus | Bacillus chittonlyticus | 113452003 |
| BACCIR | Bacillus circulans | Bacillus circulans | 71812004 |
| BACCIRG | Bacillus circulans group | Bacillus circulans group | 413619002 |
| BCLARK | Bacillus clarkii | Bacillus clarkii | 113453008 |
| BCLAUS | Bacillus clausii | Bacillus clausii | 113454002 |
| BCOHN | Bacillus cohnii | Bacillus cohnii | 113455001 |
| BDECI | Bacillus decisifrondis | Bacillus decisifrondis | 445081000 |
| BDECO | Bacillus decolorationis | Bacillus decolorationis | 429116000 |
| BDIPS | Bacillus dipsosauri | Bacillus dipsosauri | 113456000 |
| BEHIM | Bacillus ehimensis | Bacillus ehimensis | 113457009 |
| BFARR | Bacillus farraginis | Bacillus farraginis | 429348009 |
| BFAST | Bacillus fastidiosus | Bacillus fastidiosus | 35784003 |
| BACFIR | Bacillus firmus | Bacillus firmus | 13430008 |
| BFORD | Bacillus fordii | Bacillus fordii | 417062004 |
| BFORT | Bacillus fortis | Bacillus fortis | 427859002 |
| BFUMA | Bacillus fumarioli | Bacillus fumarioli | 429118004 |
| BFUNI | Bacillus funiculus | Bacillus funiculus | 428387004 |
| BGALA | Bacillus galactosidilyticus | Bacillus galactosidilyticus | 427864003 |
| BGIBS | Bacillus gibsonii | Bacillus gibsonii | 113460002 |
| BGORN | Bacillus gornadae | Bacillus gornadae | 29100001 |
| BHALM | Bacillus halmapalus | Bacillus halmapalus | 113462005 |
| BHALDF | Bacillus halodenitrificans | Bacillus halodenitrificans | 113464006 |
| BHALDR | Bacillus halodurans | Bacillus halodurans | 113465007 |
| BHORI | Bacillus horikoshii | Bacillus horikoshii | 113467004 |
| BHORT | Bacillus horti | Bacillus horti | 429138003 |
| BHUMI | Bacillus humi | Bacillus humi | 429139006 |
| BHWAJ | Bacillus hwajinpoensis | Bacillus hwajinpoensis | 429035002 |
| BIDRI | Bacillus idriensis | Bacillus idriensis | 447040007 |
| BINDI | Bacillus indicus | Bacillus indicus | 429145003 |
| BINFA | Bacillus infantis | Bacillus infantis | 432698009 |
| BINFE | Bacillus infernus | Bacillus infernus | 113468009 |
| BKAUS | Bacillus kaustophilus | Bacillus kaustophilus | 113469001 |
| BKORL | Bacillus korlensis | Bacillus korlensis | 447041006 |
| BKRUL | Bacillus krulwichiae | Bacillus krulwichiae | 429126007 |
| BACLEN | Bacillus lentus | Bacillus lentus | 3020006 |
| BACLIC | Bacillus licheniformis | Bacillus licheniformis | 74867004 |
| BLUCI | Bacillus luciferensis | Bacillus luciferensis | 429675009 |
| BMACR | Bacillus macroides | Bacillus macroides | 413620008 |
| BMACY | Bacillus macyae | Bacillus macyae | 429206000 |
| BMARS | Bacillus marisflavi | Bacillus marisflavi | 424425003 |
| BMETH | Bacillus methanolicus | Bacillus methanolicus | 113471001 |
| BMOJA | Bacillus mojavensis | Bacillus mojavensis | 113472008 |
| BMURA | Bacillus muralis | Bacillus muralis | 428576008 |
| BMYCO | Bacillus mycoides | Bacillus mycoides | 33900003 |
| BNEAL | Bacillus nealsonii | Bacillus nealsonii | 428577004 |
| BNIAC | Bacillus niacini | Bacillus niacini | 429747001 |
| BNOVA | Bacillus novalis | Bacillus novalis | 429212005 |
| BOKUH | Bacillus okuhidensis | Bacillus okuhidensis | 429468004 |
| BOLER | Bacillus oleronius | Bacillus oleronius | 113487003 |
| BPAST | Bacillus pasteurii | Bacillus pasteurii | 43131009 |
| BPATA | Bacillus patagoniensis | Bacillus patagoniensis | 428962001 |
| BPOCH | Bacillus pocheonensis | Bacillus pocheonensis | 442283002 |
| BPSDL | Bacillus pseudalcaliphilus | Bacillus pseudalcaliphilus | 113474009 |
| BPSDF | Bacillus pseudofirmus | Bacillus pseudofirmus | 113475005 |
| BPSMY | Bacillus pseudomycoides | Bacillus pseudomycoides | 429077002 |
| BPSYP | Bacillus psychrophilus | Bacillus psychrophilus | 1810009 |
| BPSYS | Bacillus psychrosaccharolyticus | Bacillus psychrosaccharolyticus | 113476006 |
| BPULV | Bacillus pulvifaciens | Bacillus pulvifaciens | 112274002 |
| BACPUM | Bacillus pumilus | Bacillus pumilus | 16580009 |
| BSALE | Bacillus salexigens | Bacillus salexigens | 113478007 |
| BSALI | Bacillus saliphilus | Bacillus saliphilus | 428588005 |
| BSELE | Bacillus selenitireducens | Bacillus selenitireducens | 428966003 |
| BSHAC | Bacillus shackletonii | Bacillus shackletonii | 429221006 |
| BSIMP | Bacillus simplex | Bacillus simplex | 113479004 |
| BSIRA | Bacillus siralis | Bacillus siralis | 428972003 |
| BACSM | Bacillus smithii | Bacillus smithii | 84789000 |
| BSONO | Bacillus sonorensis | Bacillus sonorensis | 429472000 |
| BSPOR | Bacillus sporothermodurans | Bacillus sporothermodurans | 113480001 |
| BSU | Bacillus subtilis | Bacillus subtilis | 83512007 |
| BACSUG | Bacillus subtilis group | Bacillus subtilis group | 413621007 |
| BSSP | Bacillus subtilis spizizenii | Bacillus subtilis spizizenii | 427760003 |
| BSS | Bacillus subtilis subtilis | Bacillus subtilis subtilis | 427764007 |
| BTEQU | Bacillus tequilensis | Bacillus tequilensis | 447069003 |
| BTHMN | Bacillus thermantarcticus | Bacillus thermantarcticus | 429760007 |
| BTHMM | Bacillus thermoamylovorans | Bacillus thermoamylovorans | 113481002 |
| BTHMC | Bacillus thermocatenulatus | Bacillus thermocatenulatus | 113482009 |
| BTHMCL | Bacillus thermocloacae | Bacillus thermocloacae | 113483004 |
| BACTHU | Bacillus thuringiensis | Bacillus thuringiensis | 64840009 |
| BTUSC | Bacillus tusciae | Bacillus tusciae | 34498004 |
| BVALL | Bacillus vallismortis | Bacillus vallismortis | 113484005 |
| BVEDD | Bacillus vedderi | Bacillus vedderi | 113485006 |
| BVELE | Bacillus velezensis | Bacillus velezensis | 429488000 |
| BVIET | Bacillus vietnamensis | Bacillus vietnamensis | 429229008 |
| BVIRE | Bacillus vireti | Bacillus vireti | 429230003 |
| BWEIH | Bacillus weihenstephanensis | Bacillus weihenstephanensis | 413622000 |
| BACNANT | Bacillus, not B. anthracis | Bacillus species not Bacillus anthracis | 413352001 |
| BREVISP | Brevibacillus | Brevibacillus | 114114002 |
| BREVIAGRI | Brevibacillus agri | Brevibacillus agri | 114115001 |
| BREBRE | Brevibacillus brevis | Brevibacillus brevis | 114117009 |
| BREVICENT | Brevibacillus centrosporus | Brevibacillus centrosporus | 114118004 |
| BRELAT | Brevibacillus laterosporus | Brevibacillus laterosporus | 114121002 |
| BREVIPARA | Brevibacillus parabrevis | Brevibacillus parabrevis | 114122009 |
| BRVSP | Brevibacterium | Brevibacterium | 3603008 |
| BRVCAS | Brevibacterium casei | Brevibacterium casei | 6405003 |
| BRVEPI | Brevibacterium epidermidis | Brevibacterium epidermidis | 57301000 |
| BRVLINE | Brevibacterium linens | Brevibacterium linens | 112278004 |
| BRVLUTE | Brevibacterium luteolum | Brevibacterium luteolum | 409826000 |
| BRVMCBR | Brevibacterium mcbrellneri | Brevibacterium mcbrellneri | 431099002 |
| BRVBRAVE | Brevibacterium ravenspurgense | Brevibacterium ravenspurgense | 446348002 |
| BRVSANG | Brevibacterium sanguinis | Brevibacterium sanguinis | 431681006 |
| CELLSP | Cellulomonas | Cellulomonas | 114188006 |
| CELLHOMI | Cellulomonas hominis | Cellulomonas hominis | 114196001 |
| CELLHUMI | Cellulomonas humilata | Cellulomonas humilata | 431580002 |
| CELLUSP | Cellulosimicrobium | Cellulosimicrobium | 409860007 |
| CELLUCELL | Cellulosimicrobium cellulans | Cellulosimicrobium cellulans | 409862004 |
| CORGN | Corynebacterium | Corynebacterium | 77086004 |
| CORACC | Corynebacterium accolens | Corynebacterium accolens | 243254006 |
| CORA | Corynebacterium afermentans | Corynebacterium afermentans | 113608003 |
| CORAMM | Corynebacterium ammoniagenes | Corynebacterium ammoniagenes | 113607008 |
| CORAMY | Corynebacterium amycolatum | Corynebacterium amycolatum | 113611002 |
| CORAPP | Corynebacterium appendicis | Corynebacterium appendicis | 423659000 |
| CORAQL | Corynebacterium aquilae | Corynebacterium aquilae | 424012003 |
| CORARG | Corynebacterium argentoratense | Corynebacterium argentoratense | 417499006 |
| CORATY | Corynebacterium atypicum | Corynebacterium atypicum | 424209005 |
| CORAR | Corynebacterium aurimucosum | Corynebacterium aurimucosum | 385503009 |
| CORARS | Corynebacterium auris | Corynebacterium auris | 413925000 |
| CORARSC | Corynebacterium auriscanis | Corynebacterium auriscanis | 424389005 |
| CORBET | Corynebacterium beticola | Corynebacterium beticola | 432270000 |
| CORBO | Corynebacterium bovis | Corynebacterium bovis | 44885001 |
| CORCAL | Corynebacterium callunae | Corynebacterium callunae | 88866008 |
| CORCAM | Corynebacterium camporealensis | Corynebacterium camporealensis | 424560007 |
| CORCAP | Corynebacterium capitovis | Corynebacterium capitovis | 424721008 |
| CORCAS | Corynebacterium casei | Corynebacterium casei | 424906005 |
| CORCSP | Corynebacterium caspium | Corynebacterium caspium | 422791000 |
| CORCIC | Corynebacterium ciconiae | Corynebacterium ciconiae | 424459004 |
| CORCON | Corynebacterium confusum | Corynebacterium confusum | 417134008 |
| CORCOY | Corynebacterium coyleae | Corynebacterium coyleae | 116461008 |
| CORCY | Corynebacterium cystitidis | Corynebacterium cystitidis | 50169005 |
| CORDUR | Corynebacterium durum | Corynebacterium durum | 413926004 |
| COREFF | Corynebacterium efficiens | Corynebacterium efficiens | 423379003 |
| CORFAL | Corynebacterium falsenii | Corynebacterium falsenii | 116389005 |
| CORFEL | Corynebacterium felinum | Corynebacterium felinum | 423568009 |
| CORFVS | Corynebacterium flavescens | Corynebacterium flavescens | 47802009 |
| CORFRE | Corynebacterium freneyi | Corynebacterium freneyi | 416914001 |
| CORGEN | Corynebacterium genitalium | Corynebacterium genitalium | 243260006 |
| CORGLA | Corynebacterium glaucum | Corynebacterium glaucum | 432808003 |
| CORGLU | Corynebacterium glucuronolyticum | Corynebacterium glucuronolyticum | 413927008 |
| CORGL | Corynebacterium glutamicum | Corynebacterium glutamicum | 89651003 |
| CORHAL | Corynebacterium halotolerans | Corynebacterium halotolerans | 424529007 |
| CORIMI | Corynebacterium imitans | Corynebacterium imitans | 413928003 |
| CORJK | Corynebacterium jeikeium | Corynebacterium jeikeium | 63410006 |
| CORKRO | Corynebacterium kroppenstedtii | Corynebacterium kroppenstedtii | 363777001 |
| CORKU | Corynebacterium kutscheri | Corynebacterium kutscheri | 88575000 |
| CORLIP | Corynebacterium lipophiloflavum | Corynebacterium lipophiloflavum | 416863001 |
| CORMAC | Corynebacterium macginleyi | Corynebacterium macginleyi | 363772007 |
| CORMAN | Corynebacterium manihot | Corynebacterium manihot | 243261005 |
| CORMSL | Corynebacterium massiliense | Corynebacterium massiliense | 446285007 |
| CORMST | Corynebacterium mastitidis | Corynebacterium mastitidis | 424709009 |
| CORMA | Corynebacterium matruchotii | Corynebacterium matruchotii | 89922004 |
| CORMED | Corynebacterium mediolanum | Corynebacterium mediolanum | 243262003 |
| CORMI | Corynebacterium minutissimum | Corynebacterium minutissimum | 83594007 |
| CORMUC | Corynebacterium mucifaciens | Corynebacterium mucifaciens | 413929006 |
| CORMRS | Corynebacterium murisepticum | Corynebacterium murisepticum | 302583000 |
| CORMY | Corynebacterium mycetoides | Corynebacterium mycetoides | 58879005 |
| CORNEP | Corynebacterium nephridii | Corynebacterium nephridii | 243263008 |
| CORPHO | Corynebacterium phocae | Corynebacterium phocae | 116462001 |
| CORPI | Corynebacterium pilosum | Corynebacterium pilosum | 81368007 |
| CORPRO | Corynebacterium propinquum | Corynebacterium propinquum | 113612009 |
| CORPD | Corynebacterium pseudodiphtheriticum | Corynebacterium pseudodiphtheriticum | 12050008 |
| CORPST | Corynebacterium pseudogenitalium | Corynebacterium pseudogenitalium | 243264002 |
| CORPS | Corynebacterium pseudotuberculosis | Corynebacterium pseudotuberculosis | 55084001 |
| CORPSM | Corynebacterium pseudotuberculostearicum | Corynebacterium pseudotuberculostearicum | 416570006 |
| CORPYRU | Corynebacterium pyruviciproducens | Corynebacterium pyruviciproducens | 450383002 |
| CORRE | Corynebacterium renale | Corynebacterium renale | 62000003 |
| CORRES | Corynebacterium resistens | Corynebacterium resistens | 442804004 |
| CORRIE | Corynebacterium riegelii | Corynebacterium riegelii | 131241009 |
| CORRUB | Corynebacterium rubrum | Corynebacterium rubrum | 243265001 |
| CORSIM | Corynebacterium simulans | Corynebacterium simulans | 416655000 |
| CORSIN | Corynebacterium singulare | Corynebacterium singulare | 422898002 |
| CORSPI | Corynebacterium sphenisci | Corynebacterium sphenisci | 422705004 |
| CORSPM | Corynebacterium spheniscorum | Corynebacterium spheniscorum | 432362008 |
| CORST | Corynebacterium striatum | Corynebacterium striatum | 63815007 |
| CORSUI | Corynebacterium suicordis | Corynebacterium suicordis | 423784007 |
| CORSUN | Corynebacterium sundsvallense | Corynebacterium sundsvallense | 425307003 |
| CORTEN | Corynebacterium tenuis | Corynebacterium tenuis | 72243003 |
| CORTER | Corynebacterium terpenotabidum | Corynebacterium terpenotabidum | 422496009 |
| CORTES | Corynebacterium testudinoris | Corynebacterium testudinoris | 424212008 |
| CORTHO | Corynebacterium thomssenii | Corynebacterium thomssenii | 416311000 |
| CORTUB | Corynebacterium tuberculostearicum | Corynebacterium tuberculostearicum | 416356007 |
| CORTUSC | Corynebacterium tuscaniense | Corynebacterium tuscaniense | 450409000 |
| CORUL | Corynebacterium ulcerans | Corynebacterium ulcerans | 103428000 |
| CORUR | Corynebacterium urealyticum | Corynebacterium urealyticum | 113613004 |
| CORURE | Corynebacterium ureicelerivorans | Corynebacterium ureicelerivorans | 429443001 |
| CORVAR | Corynebacterium variabile | Corynebacterium variabile | 11575001 |
| CORVIS | Corynebacterium viscosum | Corynebacterium viscosum | 243266000 |
| CORVIT | Corynebacterium vitaeruminis | Corynebacterium vitaeruminis | 116390001 |
| CORXE | Corynebacterium xerosis | Corynebacterium xerosis | 27101006 |
| CORNCD | Corynebacterium, not C. diphtheriae | Corynebacterium species, not Corynebacterium diphtheriae | 413931002 |
| CORNCJ | Corynebacterium, not C. jeikeium | Corynebacterium species not Corynebacterium jeikeium | 413930001 |
| CORGPB | coryneform gram positive bacilli | Coryneform bacteria | 116442009 |
| CUTISP | Cutibacterium | Cutibacterium | 763051006 |
| PRPAC | Cutibacterium acnes | Cutibacterium acnes | 39473003 |
| PROAV | Cutibacterium avidum | Cutibacterium avidum | 47473006 |
| PROGR | Cutibacterium granulosum | Cutibacterium granulosum | 87755005 |
| DERMASP | Dermabacter | Dermabacter | 413976002 |
| DERMAHOM | Dermabacter hominis | Dermabacter hominis | 413977006 |
| DERMSP | Dermacoccus | Dermacoccus | 115142006 |
| DENISH | Dermacoccus nishinomiyaensis | Dermacoccus nishinomiyaensis | 113776005 |
| DIPTH | Diphtheroids | Diphtheroids | 54642001 |
| DIPTHAE | Diphtheroids, aerobic | Aerobic diphtheroids | 44661006 |
| DIPTHAN | Diphtheroids, anaerobic | Anaerobic diphtheroids | 26481006 |
| EXIGSP | Exiguobacterium | Exiguobacterium | 114178005 |
| EXIGACET | Exiguobacterium acetylicum | Exiguobacterium acetylicum | 114179002 |
| GORDSP | Gordonia | Gordonia | 115143001 |
| GORDBRON | Gordonia bronchialis | Gordonia bronchialis | 113646005 |
| GORDOTIT | Gordonia otitidis | Gordonia otitidis | 443659007 |
| GORDPOLY | Gordonia polyisoprenivorans | Gordonia polyisoprenivorans | 420096009 |
| GORDRUBR | Gordonia rubripertincta | Gordonia rubripertincta | 113647001 |
| GORDSPUT | Gordonia sputi | Gordonia sputi | 113648006 |
| GORDTERR | Gordonia terrae | Gordonia terrae | 113649003 |
| JANISP | Janibacter | Janibacter | 409828004 |
| JANIHOYL | Janibacter hoylei | Janibacter hoylei | 698246003 |
| KOCSP | Kocuria | Kocuria | 414328004 |
| KOCKRI | Kocuria kristinae | Kocuria kristinae | 113772007 |
| KOCROS | Kocuria rosea | Kocuria rosea | 113773002 |
| KOCVAR | Kocuria varians | Kocuria varians | 113774008 |
| KYTSP | Kytococcus | Kytococcus | 115141004 |
| KYSED | Kytococcus sedentarius | Kytococcus sedentarius | 113775009 |
| LEIFSP | Leifsonia | Leifsonia | 414590004 |
| LEIFAQUA | Leifsonia aquatica | Leifsonia aquatica | 414591000 |
| LEIFXYLI | Leifsonia xyli | Leifsonia xyli | 414596005 |
| MICRSP | Microbacterium | Microbacterium | 114199008 |
| MICRARBO | Microbacterium arborescens | Microbacterium arborescens | 414699006 |
| MICRHYDR | Microbacterium hydrocarbonoxydans | Microbacterium hydrocarbonoxydans | 414707005 |
| MICRIMPE | Microbacterium imperiale | Microbacterium imperiale | 114203008 |
| MICRLACT | Microbacterium lacticum | Microbacterium lacticum | 114204002 |
| MICRLIQU | Microbacterium liquefaciens | Microbacterium liquefaciens | 114063000 |
| MICRMARI | Microbacterium maritypicum | Microbacterium maritypicum | 414710003 |
| MICROXYD | Microbacterium oxydans | Microbacterium oxydans | 414713001 |
| MICRPARA | Microbacterium paraoxydans | Microbacterium paraoxydans | 414714007 |
| MICRRESI | Microbacterium resistens | Microbacterium resistens | 414716009 |
| MICG | Micrococcus | Micrococcus | 52864008 |
| MICANT | Micrococcus antarcticus | Micrococcus antarcticus | 416555005 |
| MICLUT | Micrococcus luteus | Micrococcus luteus | 48299003 |
| MICLYL | Micrococcus lylae | Micrococcus lylae | 61376003 |
| MICNIS | Micrococcus nishinomiyaensis | Micrococcus nishinomiyaensis | 67084000 |
| OERSP | Oerskovia | Oerskovia | 81194007 |
| PAESP | Paenibacillus | Paenibacillus | 114086007 |
| PAEAGAR | Paenibacillus agaridevorans | Paenibacillus agaridevorans | 429907003 |
| PAEALV | Paenibacillus alvei | Paenibacillus alvei | 114088008 |
| BEDAP | Paenibacillus edaphicus | Paenibacillus edaphicus | 8501000146105 |
| PAELARV | Paenibacillus larvae | Paenibacillus larvae | 114098002 |
| PAELENT | Paenibacillus lentimorbus | Paenibacillus lentimorbus | 414989004 |
| PAEMAC | Paenibacillus macerans | Paenibacillus macerans | 114101003 |
| PAEPABU | Paenibacillus pabuli | Paenibacillus pabuli | 114103000 |
| PAEPOL | Paenibacillus polymyxa | Paenibacillus polymyxa | 114105007 |
| PAEPOPI | Paenibacillus popilliae | Paenibacillus popilliae | 414990008 |
| PAEPROV | Paenibacillus provencensis | Paenibacillus provencensis | 438962008 |
| PAETHIA | Paenibacillus thiaminolyticus | Paenibacillus thiaminolyticus | 114106008 |
| PAEURIN | Paenibacillus urinalis | Paenibacillus urinalis | 445240003 |
| PAEVALI | Paenibacillus validus | Paenibacillus validus | 114107004 |
| PRPG | Propionibacterium | Propionibacterium | 34844008 |
| PRPAU | Propionibacterium australiense | Propionibacterium australiense | 433037008 |
| PRPCY | Propionibacterium cyclohexanicum | Propionibacterium cyclohexanicum | 113916009 |
| PRPF | Propionibacterium freudenreichii | Propionibacterium freudenreichii | 23871008 |
| PRPPR | Propionibacterium prionicus | Propionibacterium prionicus | 113919002 |
| PROPISP | Propioniferax | Propioniferax | 114055001 |
| RHOSP | Rhodococcus | Rhodococcus | 34534002 |
| RHOERYT | Rhodococcus erythropolis | Rhodococcus erythropolis | 22478004 |
| RHOFASC | Rhodococcus fascians | Rhodococcus fascians | 28901005 |
| RHOGLOB | Rhodococcus globerulus | Rhodococcus globerulus | 44348004 |
| RHOGORD | Rhodococcus gordoniae | Rhodococcus gordoniae | 430885009 |
| RHORHOD | Rhodococcus rhodochrous | Rhodococcus rhodochrous | 54379008 |
| ROSE | Roseomonas | Roseomonas | 115159001 |
| ROSECERV | Roseomonas cervicalis | Roseomonas cervicalis | 113711006 |
| ROSEGILA | Roseomonas gilardii | Roseomonas gilardii | 113710007 |
| ROSEMUCO | Roseomonas mucosa | Roseomonas mucosa | 415351006 |
| ROTSP | Rothia | Rothia | 90163001 |
| ROTAERI | Rothia aeria | Rothia aeria | 417029007 |
| ROTDE | Rothia dentocariosa | Rothia dentocariosa | 35379007 |
| ROTHMU | Rothia mucilaginosa | Rothia mucilaginosa | 416544005 |
| RUMMSP | Rummeliibacillus | Rummeliibacillus | 450458001 |
| RUMMPYCN | Rummeliibacillus pycnus | Rummeliibacillus pycnus | 450459009 |
| STRBA | Small-colony-forming beta-hemolytic group A streptococci | Small-colony-forming beta-hemolytic group A streptococci | 415534000 |
| STRBC | Small-colony-forming beta-hemolytic group C streptococci | Small-colony-forming beta-hemolytic group C streptococci | 415535004 |
| STRBG | Small-colony-forming beta-hemolytic group G streptococci | Small-colony-forming beta-hemolytic group G streptococci | 415536003 |
| SOLISP | Solibacillus | Solibacillus | 699375003 |
| BSILV | Solibacillus silvestris | Solibacillus silvestris | 429471007 |
| STAARL | Staphylococcus arlettae | Staphylococcus arlettae | 72465001 |
| STAAR | Staphylococcus auricularis | Staphylococcus auricularis | 45654003 |
| STACS | Staphylococcus capitis | Staphylococcus capitis | 40347003 |
| STACC | Staphylococcus capitis capitis | Staphylococcus capitis ss capitis | 113962001 |
| STACU | Staphylococcus capitis urealyticus | Staphylococcus capitis subspecies urealyticus | 103492009 |
| STACAP | Staphylococcus caprae | Staphylococcus caprae | 76969000 |
| STACAR | Staphylococcus carnosus | Staphylococcus carnosus | 9784002 |
| STACRC | Staphylococcus carnosus carnosus | Staphylococcus carnosus carnosus | 438905007 |
| STACUT | Staphylococcus carnosus utilis | Staphylococcus carnosus utilis | 438733001 |
| STACHR | Staphylococcus chromogenes | Staphylococcus chromogenes | 56629004 |
| STACO | Staphylococcus cohnii | Staphylococcus cohnii | 66872005 |
| STASCO | Staphylococcus cohnii cohnii | Staphylococcus cohnii ss cohnii | 113963006 |
| STASUR | Staphylococcus cohnii urealyticus | Staphylococcus cohnii subspecies urealyticus | 103493004 |
| STACON | Staphylococcus condimenti | Staphylococcus condimenti | 439185000 |
| SE | Staphylococcus epidermidis | Staphylococcus epidermidis | 60875001 |
| STAEQ | Staphylococcus equorum | Staphylococcus equorum | 28217008 |
| STAEE | Staphylococcus equorum equorum | Staphylococcus equorum equorum | 438005005 |
| STAEL | Staphylococcus equorum linens | Staphylococcus equorum linens | 438164005 |
| STAFE | Staphylococcus felis | Staphylococcus felis | 113965004 |
| STAGA | Staphylococcus gallinarum | Staphylococcus gallinarum | 8056006 |
| STAHA | Staphylococcus haemolyticus | Staphylococcus haemolyticus | 83452006 |
| STAHO | Staphylococcus hominis | Staphylococcus hominis | 44827007 |
| STAHOM | Staphylococcus hominis hominis | Staphylococcus hominis hominis | 432505008 |
| STANOV | Staphylococcus hominis novobiosepticus | Staphylococcus hominis novobiosepticus | 438202004 |
| STAKLO | Staphylococcus kloosii | Staphylococcus kloosii | 53227003 |
| STALU | Staphylococcus lugdunensis | Staphylococcus lugdunensis | 103494005 |
| STAMUS | Staphylococcus muscae | Staphylococcus muscae | 113969005 |
| STANEP | Staphylococcus nepalensis | Staphylococcus nepalensis | 438681002 |
| STAPAS | Staphylococcus pasteuri | Staphylococcus pasteuri | 103495006 |
| STAPETT | Staphylococcus pettenkoferi | Staphylococcus pettenkoferi | 428731005 |
| STAPIS | Staphylococcus piscifermentans | Staphylococcus piscifermentans | 113970006 |
| STASL | Staphylococcus saccharolyticus | Staphylococcus saccharolyticus | 41103009 |
| STASA | Staphylococcus saprophyticus | Staphylococcus saprophyticus | 19743000 |
| STASB | Staphylococcus saprophyticus bovis | Staphylococcus saprophyticus ss bovis | 113972003 |
| STASAP | Staphylococcus saprophyticus saprophyticus | Staphylococcus saprophyticus ss saprophyticus | 113973008 |
| STASCH | Staphylococcus schleiferi schleiferi | Staphylococcus schleiferi subsp. schleiferi | 103496007 |
| STASI | Staphylococcus simulans | Staphylococcus simulans | 52122005 |
| STASU | Staphylococcus succinus | Staphylococcus succinus | 438729008 |
| STASUC | Staphylococcus succinus casei | Staphylococcus succinus casei | 440815004 |
| STASUS | Staphylococcus succinus succinus | Staphylococcus succinus succinus | 441130000 |
| STAWA | Staphylococcus warneri | Staphylococcus warneri | 63550006 |
| STAXY | Staphylococcus xylosus | Staphylococcus xylosus | 32737005 |
| CNS | Staphylococcus, coagulase negative | Staphylococcus, coagulase negative | 116197008 |
| STRALAC | Streptococcus alactolyticus | Streptococcus alactolyticus | 34228005 |
| STRVN | Streptococcus anginosus | Streptococcus anginosus | 44304009 |
| STRANG | Streptococcus anginosus group | Streptococcus anginosus group | 415597009 |
| STRAC | Streptococcus anginosus-constellatus | Streptococcus anginosus-constellatus | 70160008 |
| STRAUS | Streptococcus australis | Streptococcus australis | 415598004 |
| STRBOVI | Streptococcus bovis group | Streptococcus bovis group | 415599007 |
| STRVC | Streptococcus constellatus | Streptococcus constellatus | 76199005 |
| STRCC | Streptococcus constellatus constellatus | Streptococcus constellatus subspecies constellatus | 415601009 |
| STRCP | Streptococcus constellatus pharyngis | Streptococcus constellatus subspecies pharyngis | 415602002 |
| STRCRC | Streptococcus criceti | Streptococcus criceti | 34838009 |
| STRCR | Streptococcus cristatus | Streptococcus cristatus | 113980005 |
| STRDENT | Streptococcus dentisani | Streptococcus dentisani | 708952006 |
| STRD | Streptococcus downei | Streptococcus downei | 12704003 |
| STRDE | Streptococcus equinus | Streptococcus equinus | 72646003 |
| STRF | Streptococcus ferus | Streptococcus ferus | 10049004 |
| STRGALL | Streptococcus gallolyticus | Streptococcus gallolyticus | 113985000 |
| STRGALGAL | Streptococcus gallolyticus gallolyticus | Streptococcus gallolyticus subspecies gallolyticus | 421544007 |
| STRGALMAC | Streptococcus gallolyticus macedonicus | Streptococcus gallolyticus subspecies macedonicus | 421004005 |
| STRGALPAS | Streptococcus gallolyticus pasteurianus | Streptococcus gallolyticus subspecies pasteurianus | 421253004 |
| STRVG | Streptococcus gordonii | Streptococcus gordonii | 113986004 |
| STRHYOV | Streptococcus hyovaginalis | Streptococcus hyovaginalis | 113988003 |
| STRINFA | Streptococcus infantarius | Streptococcus infantarius | 415603007 |
| STRINFINF | Streptococcus infantarius infantarius | Streptococcus infantarius subspecies infantarius | 432506009 |
| STRINF | Streptococcus infantis | Streptococcus infantis | 415604001 |
| SVI | Streptococcus intermedius | Streptococcus intermedius | 62170002 |
| STRLUTE | Streptococcus lutetiensis | Streptococcus lutetiensis | 415605000 |
| STRMACA | Streptococcus macacae | Streptococcus macacae | 59717000 |
| STRVM | Streptococcus mitis | Streptococcus mitis | 57997003 |
| STRMIT | Streptococcus mitis group | Streptococcus mitis group | 127539005 |
| STRVT | Streptococcus mutans | Streptococcus mutans | 214001 |
| STRMUG | Streptococcus mutans group | Streptococcus mutans group | 127536003 |
| STROLIG | Streptococcus oligofermentans | Streptococcus oligofermentans | 440296007 |
| STROR | Streptococcus oralis | Streptococcus oralis | 19870004 |
| STRPA | Streptococcus parasanguinis | Streptococcus parasanguinis | 113990002 |
| STRPER | Streptococcus peroris | Streptococcus peroris | 416347004 |
| STRPSEU | Streptococcus pseudopneumoniae | Streptococcus pseudopneumoniae | 420470004 |
| STRRT | Streptococcus ratti | Streptococcus ratti | 10667007 |
| STRVS | Streptococcus salivarius | Streptococcus salivarius | 39888004 |
| STRSAG | Streptococcus salivarius group | Streptococcus salivarius group | 127537007 |
| STRSLV | Streptococcus salivarius salivarius | Streptococcus salivarius subsp. salivarius | 84684000 |
| STRSAL | Streptococcus salivarius thermophilus | Streptococcus salivarius subsp. thermophilus | 23310006 |
| SVS | Streptococcus sanguinis | Streptococcus sanguinis | 113993000 |
| STRSG | Streptococcus sanguinis group | Streptococcus sanguinis group | 115658008 |
| STRSINE | Streptococcus sinensis | Streptococcus sinensis | 438909001 |
| STRSO | Streptococcus sobrinus | Streptococcus sobrinus | 43777007 |
| STRTIGU | Streptococcus tigurinus | Streptococcus tigurinus | 699479005 |
| STRVE | Streptococcus vestibularis | Streptococcus vestibularis | 113998009 |
| GFS | Streptococcus, group F | Streptococcus, group F | 83503005 |
| TRUESP | Trueperella | Trueperella | 704974007 |
| TRUEBERN | Trueperella bernardiae | Trueperella bernardiae | 704975008 |
| ARCPY | Trueperella pyogenes | Trueperella pyogenes | 258933002 |
| TSUKSP | Tsukamurella | Tsukamurella | 115135006 |
| TSUKINCH | Tsukamurella inchonensis | Tsukamurella inchonensis | 113509000 |
| TSUKPAUR | Tsukamurella paurometabola | Tsukamurella paurometabola | 113510005 |
| TSUKPULM | Tsukamurella pulmonis | Tsukamurella pulmonis | 113511009 |
| TSUKSTRA | Tsukamurella strandjordii | Tsukamurella strandjordii | 417316004 |
| TSUKTYRO | Tsukamurella tyrosinosolvens | Tsukamurella tyrosinosolvens | 113512002 |
| TURISP | Turicella | Turicella | 415762009 |
| VIRSP | Virgibacillus | Virgibacillus | 114108009 |
| VIRPAN | Virgibacillus pantothenticus | Virgibacillus pantothenticus | 114109001 |
| SVU | Viridans group streptococci | Streptococcus, viridans group | 31703004 |

**Supplementary Table 2** List of covariates used for calculation of propensity scores for inverse probability weighting and for covariate adjustment in generalized linear model

| Age, years |
| --- |
| Sex |
| Year of Admission |
| Days from ICU admission to index Culture |
| Type of ICU (medical, surgical, emergency) |
| Endocrine, Nutritional, and Metabolic Diseases |
| Diabetes Mellitus |
| Chronic Kidney Disease |
| Diseases of the Digestive System |
| Hypertensive Disease |
| Ischemic Heart Disease |
| Cerebrovascular Disease |
| Arterial Disease |
| Neoplasms |
| Diseases of the Respiratory System |
| Diseases of the Nervous System |
| Diseases of the Skin and Subcutaneous Tissue |
| Mechanical Ventilation Prior to Index Culture |
| ECMO at Index Culture |
| SOFA Score Category |
| PaO2/FiO2 Category |
| Fever |
| Vasopressor Use Prior to Index Culture |
| Inotrope Use Prior to Index Culture |
| Antibiotic Use Prior to Index Culture |
| Corticosteroid Use Prior to Index Culture |
| Immunosuppressant Use Prior to Index Culture |
| Procalcitonin at time of index culture |
| Community-acquired versus hospital-acquired infection (index culture < or ≥48h after hospital admission) |

*The categorization of continuous variables is identical to those presented in Table 1.

**Supplementary Table 3** Baseline characteristics in common commensal-free cohort

|  | **Overall** | **Monomicrobial** | **Polymicrobial** | **p** |
| --- | --- | --- | --- | --- |
| n | 1669 | 1501 | 168 |  |
| Age, years (median [IQR]) | 61.0 [51.0, 71.0] | 61.0 [51.0, 70.0] | 62.5 [51.0, 73.0] | 0.357 |
| Age category (%) |  |  |  | 0.681 |
| 49 years or younger | 288 (18.3) | 261 (18.4) | 27 (17.3) |  |
| 50 to 64 years | 597 (38.0) | 541 (38.2) | 56 (35.9) |  |
| 65 to 74 years | 413 (26.3) | 374 (26.4) | 39 (25.0) |  |
| 74 to 79 years | 160 (10.2) | 140 (9.9) | 20 (12.8) |  |
| 80 years or older | 114 (7.3) | 100 (7.1) | 14 (9.0) |  |
| Female sex (%) | 614 (36.8) | 560 (37.3) | 54 (32.1) | 0.218 |
| Geographical origin (%) |  |  |  | 0.497 |
| Africa | 7 (0.4) | 6 (0.4) | 1 (0.6) |  |
| Europe | 1437 (86.1) | 1297 (86.4) | 140 (83.3) |  |
| Middle East or Asia | 27 (1.6) | 22 (1.5) | 5 (3.0) |  |
| North or South America | 5 (0.3) | 5 (0.3) | 0 (0.0) |  |
| Days from ICU admission to index culture (median [IQR]) | 5.0 [1.0, 16.0] | 5.0 [1.0, 16.0] | 3.0 [0.0, 15.0] | 0.096 |
| Days from ICU admission to index culture (category) |  |  |  | 0.156 |
| Day 1 to 2 | 708 (42.4) | 627 (41.8) | 81 (48.2) |  |
| Day 3 to 7 | 264 (15.8) | 237 (15.8) | 27 (16.1) |  |
| Day 8 to 14 | 250 (15.0) | 234 (15.6) | 16 (9.5) |  |
| Day 15 or later | 447 (26.8) | 403 (26.8) | 44 (26.2) |  |
| Number of bacterial pathogens in index culture |  |  |  | <0.001 |
| 1 | 1501 (89.9) | 1501 (100.0) | 0 (0.0) |  |
| 2 | 147 (8.8) | 0 (0.0) | 147 (87.5) |  |
| 3 | 18 (1.1) | 0 (0.0) | 18 (10.7) |  |
| 4 | 2 (0.1) | 0 (0.0) | 2 (1.2) |  |
| 5 | 1 (0.1) | 0 (0.0) | 1 (0.6) |  |
| 6 |  |  |  | 0.566 |
| Time period of admission (%) | 279 (16.7) | 251 (16.7) | 28 (16.7) |  |
| 2014/15 | 348 (20.9) | 306 (20.4) | 42 (25.0) |  |
| 2016/17 | 216 (12.9) | 201 (13.4) | 15 (8.9) |  |
| 2018/19 | 404 (24.2) | 364 (24.3) | 40 (23.8) |  |
| 2020/21 | 382 (22.9) | 343 (22.9) | 39 (23.2) |  |
| 2022/23 | 40 (2.4) | 36 (2.4) | 4 (2.4) |  |
| 2024 |  |  |  | 0.994 |
| Type of ICU (%) | 102 (6.1) | 92 (6.1) | 10 (6.0) |  |
| Emergency Medicine ICU | 916 (54.9) | 824 (54.9) | 92 (54.8) |  |
| Medical ICU | 651 (39.0) | 585 (39.0) | 66 (39.3) |  |
| Surgical ICU | 338 (20.3) | 306 (20.4) | 32 (19.0) | 0.758 |
| Endocrine, nutritional, and metabolic diseases (%) | 266 (15.9) | 235 (15.7) | 31 (18.5) | 0.408 |
| Diabetes (%) | 625 (37.4) | 565 (37.6) | 60 (35.7) | 0.685 |
| Chronic kidney disease (%) | 365 (21.9) | 331 (22.1) | 34 (20.2) | 0.659 |
| Diseases of the digestive system (%) | 1275 (76.4) | 1150 (76.6) | 125 (74.4) | 0.586 |
| Hypertensive disease (%) | 549 (32.9) | 495 (33.0) | 54 (32.1) | 0.895 |
| Ischemic heart disease (%) | 114 (6.8) | 96 (6.4) | 18 (10.7) | 0.052 |
| Cerebrovascular disease (%) | 170 (10.2) | 156 (10.4) | 14 (8.3) | 0.482 |
| Arterial disease (%) | 256 (15.3) | 223 (14.9) | 33 (19.6) | 0.129 |
| Neoplasms (%) | 386 (23.1) | 350 (23.3) | 36 (21.4) | 0.650 |
| Diseases of the respiratory system (%) | 173 (10.4) | 155 (10.3) | 18 (10.7) | 0.982 |
| Diseases of the nervous system (%) | 70 (4.2) | 58 (3.9) | 12 (7.1) | 0.071 |
| Diseases of the skin and subcutaneous tissue (%) | 386 (23.1) | 347 (23.1) | 39 (23.2) | 1.000 |
| Mechanical ventilation at time of index culture (%) | 71 (4.3) | 63 (4.2) | 8 (4.8) | 0.887 |
| Extracorporeal membrane oxygenation (ECMO) at index culture (%) | 7.0 [4.0, 11.0] | 7.0 [4.0, 11.0] | 8.0 [4.0, 10.0] | 0.826 |
| Sequential Organ Failure Assessment (SOFA) score (median [IQR]) |  |  |  | 0.850 |
| SOFA Score (%) | 341 (20.4) | 308 (20.5) | 33 (19.6) |  |
| 0 to 6 | 278 (16.7) | 251 (16.7) | 27 (16.1) |  |
| 10 or higher | 236 (14.1) | 215 (14.3) | 21 (12.5) |  |
| 7 to 9 | 814 (48.8) | 727 (48.4) | 87 (51.8) |  |
| Not available |  |  |  | 0.762 |
| PaO_2_/FiO_2_ (%) | 208 (12.5) | 186 (12.4) | 22 (13.1) |  |
| 100 mmHg or less | 370 (22.2) | 335 (22.3) | 35 (20.8) |  |
| 100 to 199 mmHg | 151 (9.0) | 138 (9.2) | 13 (7.7) |  |
| 200 to 299 mmHg | 62 (3.7) | 58 (3.9) | 4 (2.4) |  |
| 300 mmHg or more | 878 (52.6) | 784 (52.2) | 94 (56.0) |  |
| Not available |  |  |  | 0.142 |
| Fever (%) | 229 (13.7) | 199 (13.3) | 30 (17.9) |  |
| 37.5°C or less | 103 (6.2) | 93 (6.2) | 10 (6.0) |  |
| 37.6 to 37.9°C | 384 (23.0) | 350 (23.3) | 34 (20.2) |  |
| 38.0 to 39.9°C | 29 (1.7) | 23 (1.5) | 6 (3.6) |  |
| 40.0°C or more | 924 (55.4) | 836 (55.7) | 88 (52.4) |  |
| Not available | 962 (57.6) | 856 (57.0) | 106 (63.1) | 0.154 |
| Vasopressor at time of index culture (%) | 186 (11.1) | 168 (11.2) | 18 (10.7) | 0.954 |
| Inotrope at time of index culture (%) | 823 (49.3) | 746 (49.7) | 77 (45.8) | 0.385 |
| Antimicrobial treatment at time of index culture (%) | 323 (19.4) | 294 (19.6) | 29 (17.3) | 0.535 |
| Corticosteroid treatment at time of index culture (%) | 109 (6.5) | 104 (6.9) | 5 (3.0) | 0.072 |
| Immunosuppression at time of index culture (%) | 13.13 (10.6) | 13.0 (10.4) | 14.7 (11.8) | 0.053 |
| Leukocytes at time of index culture, G/L (mean (SD)) |  |  |  | 0.500 |
| Leukocytes category, G/L (%) | 179 (10.7) | 164 (10.9) | 15 (8.9) |  |
| Less than 4 | 524 (31.4) | 476 (31.7) | 48 (28.6) |  |
| 4 to 9 | 359 (21.5) | 325 (21.7) | 34 (20.2) |  |
| 10 to 13 | 558 (33.4) | 494 (32.9) | 64 (38.1) |  |
| 14 or more | 49 (2.9) | 42 (2.8) | 7 (4.2) |  |
| Not available | 16.4 (11.4) | 16.3 (11.4) | 17.3 (11.7) | 0.320 |
| C-reactive protein at index culture, mg/dL (mean (SD)) |  |  |  | 0.800 |
| C-reactive protein category, mg/dL (%) | 13 (0.8) | 12 (0.8) | 1 (0.6) |  |
| Less than 0.5 | 563 (33.7) | 512 (34.1) | 51 (30.4) |  |
| 0.5 to 9 | 490 (29.4) | 439 (29.2) | 51 (30.4) |  |
| 10 to 19 | 552 (33.1) | 494 (32.9) | 58 (34.5) |  |
| 20 or more | 51 (3.1) | 44 (2.9) | 7 (4.2) |  |
| Not available | 15.3 (26.7) | 14.9 (26.2) | 19.2 (30.2) | 0.150 |
| Procalcitonin at index culture, ng/ml (mean (SD)) |  |  |  | 0.882 |
| Procalcitonin category, ng/ml (%) | 223 (13.4) | 202 (13.5) | 21 (12.5) |  |
| Less than 0.5 | 187 (11.2) | 167 (11.1) | 20 (11.9) |  |
| 0.5 to 1.9 | 191 (11.4) | 173 (11.5) | 18 (10.7) |  |
| 02 to 9.9 | 274 (16.4) | 242 (16.1) | 32 (19.0) |  |
| 10 or higher | 794 (47.6) | 717 (47.8) | 77 (45.8) |  |
| Not available | 2.07 (2.1) | 2.07 (2.1) | 1.98 (1.9) | 0.581 |
| Creatinine at index culture, mg/dL (mean (SD)) |  |  |  | 0.907 |
| Creatinine protein category, mg/dL (%) | 587 (35.2) | 528 (35.2) | 59 (35.1) |  |
| Less than 0.9 | 308 (18.5) | 274 (18.3) | 34 (20.2) |  |
| 1.0 to 1.4 | 189 (11.3) | 173 (11.5) | 16 (9.5) |  |
| 01.5 to 1.9 | 216 (12.9) | 194 (12.9) | 22 (13.1) |  |
| 2.0 to 2.9 | 316 (18.9) | 286 (19.1) | 30 (17.9) |  |
| 3.0 or higher | 53 (3.2) | 46 (3.1) | 7 (4.2) |  |
| Not available | 807 (48.4) | 737 (49.1) | 70 (41.7) | 0.081 |
| Direct ICU admission (%) | 476 (28.5) | 428 (28.5) | 48 (28.6) | 1.000 |

**Supplementary Table 4** Primary and secondary outcomes in common commensal-free cohort

| **Group** | **N** | **Unadjusted Risk**  **Or**  **Mean (95% CI)** | **Unadjusted RD**  **Or**  **mean difference (95% CI)** | **Unadj. p** | **Adjusted Risk**  **Or**  **Mean (95% CI)** | **Adjusted RD**  **Or**  **mean difference (95% CI)** | **Adj. p** |
| --- | --- | --- | --- | --- | --- | --- | --- |
| **Total sample size** | | | | | | | |
| Monomicrobial | 1501 |  |  |  |  |  |  |
| Polymicrobial | 168 |  |  |  |  |  |  |
| **Death at 90 days** | | | | | | | |
| Monomicrobial | 512 | 34.1% (31.7–36.5%) |  |  | 34.2% (32.1–36.4%) |  |  |
| Polymicrobial | 61 | 36.3% (29.0–43.5%) | 2.2% (-5.46–9.86%) | 0.57 | 36.6% (30.1–43.1%) | 2.36% (-4.48–9.2%) | 0.5 |
| **Death at 30 days** | | | | | | | |
| Monomicrobial | 285 | 19.0% (17–21.0%) |  |  | 19.3% (17.6–21.0%) |  |  |
| Polymicrobial | 34 | 20.2% (14.2–26.3%) | 1.25% (-5.14–7.64%) | 0.7 | 19.6% (14.5–24.7%) | 0.31% (-5.06–5.67%) | 0.91 |
| **Microbiological failure (any pathogen)** | | | | | | | |
| Monomicrobial | 92 | 6.1% (4.9–7.3%) |  |  | 6.12% (4.94–7.31%) |  |  |
| Polymicrobial | 11 | 6.6% (2.8–10.3%) | 0.42% (-3.51–4.35%) | 0.83 | 4.76% (2.07–7.45%) | -1.36% (-4.31–1.59%) | 0.36 |
| **Microbiological failure (same pathogen)** | | | | | | | |
| Monomicrobial | 81 | 5.4% (4.25–6.54%) |  |  | 5.38% (4.25–6.5%) |  |  |
| Polymicrobial | 11 | 6.55% (2.81–10.29%) | 1.15% (-2.76–5.06%) | 0.56 | 5.8% (2.56–9.05%) | 0.42% (-3.02–3.87%) | 0.81 |
| **New onset ECMO at 30 days** | | | | | | | |
| Monomicrobial | 48 | 3.2% (2.31–4.09%) |  |  | 3.17% (2.38–3.96%) |  |  |
| Polymicrobial | 3 | 1.79% (-0.22–3.79%) | -1.41% (-3.6–0.78%) | 0.21 | 2.43% (0.1–4.76%) | -0.74% (-3.2–1.71%) | 0.55 |
| **New onset ARDS at 7 days** | | | | | | | |
| Monomicrobial | 263 | 17.5% (15.6–19.4%) |  |  | 17.5% (15.8–19.3%) |  |  |
| Polymicrobial | 24 | 14.3% (9.0–19.6%) | -3.24% (-8.87–2.39%) | 0.26 | 17.1% (12.4–21.8%) | -0.45% (-5.22–4.33%) | 0.85 |
| **Fever at 7 days** | | | | | | | |
| Monomicrobial | 260 | 17.3% (15.4–19.2%) |  |  | 17.5% (15.8–19.1%) |  |  |
| Polymicrobial | 29 | 17.3% (11.6–23.0%) | -0.06% (-6.09–5.97%) | 0.98 | 14.3% (10.2–18.5%) | -3.13% (-7.55–1.3%) | 0.17 |
| **Length of ICU stay (days after index culture)** | | | | | | | |
| Monomicrobial |  | 20.1 (18.9–21.3) |  |  | 20 (18.9–21.11) |  |  |
| Polymicrobial |  | 20.1 (16.5–23.6) | -0.03 (-3.78–3.72) | 0.99 | 21.2 (17.8–24.6) | 1.16 (-2.43–4.76) | 0.53 |
| **Length of hospital stay (days after index culture)** | | | | | | | |
| Monomicrobial |  | 45.8 (41.8–49.8) |  |  | 45.7 (41.8–49.7) |  |  |
| Polymicrobial |  | 41.4 (29.4–53.3) | -4.49 (-17.09–8.11) | 0.48 | 40.1 (34.6–45.6) | -5.58 (-12.23–1.06) | 0.1 |

**Supplementary Table 5** Overview of antimicrobial treatment in the overall cohort

|  | Overall | Monomicrobial | Polymicrobial | p |
| --- | --- | --- | --- | --- |
| n | 3197 | 2648 | 549 |  |
| Any antimicrobial treatment (%) | 2777 (86.9) | 2289 (86.4) | 488 (88.9) | 0.140 |
| Aminopenicillin (%) | 435 (13.6) | 367 (13.9) | 68 (12.4) | 0.396 |
| Other penicillins (%) | 53 (1.7) | 48 (1.8) | 5 (0.9) | 0.186 |
| Extended-spectrum penicillins (piperacillin-tazobactam) (%) | 1248 (39.0) | 1016 (38.4) | 232 (42.3) | 0.098 |
| 1st Gen cephalosporins (%) | 113 (3.5) | 104 (3.9) | 9 (1.6) | 0.012 |
| 2nd Gen cephalosporins (%) | 122 (3.8) | 103 (3.9) | 19 (3.5) | 0.723 |
| 3rd Gen cephalosporins (%) | 235 (7.4) | 201 (7.6) | 34 (6.2) | 0.293 |
| 4th Gen cephalosporins (%) | 290 (9.1) | 231 (8.7) | 59 (10.7) | 0.155 |
| 5th Gen cephalosporins (%) | 3 (0.1) | 2 (0.1) | 1 (0.2) | 1.000 |
| Carbapenems (%) | 1046 (32.7) | 860 (32.5) | 186 (33.9) | 0.557 |
| Fluoroquinolones (%) | 195 (6.1) | 166 (6.3) | 29 (5.3) | 0.435 |
| Aminoglycosides (%) | 213 (6.7) | 171 (6.5) | 42 (7.7) | 0.355 |
| Macrolides (%) | 480 (15.0) | 393 (14.8) | 87 (15.8) | 0.593 |
| Clindamycin (%) | 118 (3.7) | 99 (3.7) | 19 (3.5) | 0.849 |
| Tetracyclines (%) | 63 (2.0) | 52 (2.0) | 11 (2.0) | 1.000 |
| Sulfonamides (%) | 198 (6.2) | 161 (6.1) | 37 (6.7) | 0.627 |
| Glycopeptides (%) | 319 (10.0) | 253 (9.6) | 66 (12.0) | 0.093 |
| Oxazolidinones (%) | 767 (24.0) | 622 (23.5) | 145 (26.4) | 0.160 |
| Rifamycins (%) | 9 (0.3) | 8 (0.3) | 1 (0.2) | 0.968 |
| Nitroimidazoles (%) | 192 (6.0) | 155 (5.9) | 37 (6.7) | 0.486 |
| Aztreonam (%) | 100 (3.1) | 79 (3.0) | 21 (3.8) | 0.370 |
| Polymyxins (%) | 31 (1.0) | 25 (0.9) | 6 (1.1) | 0.933 |
| Fosfomycin (%) | 272 (8.5) | 221 (8.3) | 51 (9.3) | 0.524 |
| Others (%) | 119 (3.7) | 95 (3.6) | 24 (4.4) | 0.448 |

**Supplementary Table 6** Overview of antimicrobial treatment in common commensal-free cohort

|  | Overall | Monomicrobial | Polymicrobial | p |
| --- | --- | --- | --- | --- |
| n | 1669 | 1501 | 168 |  |
| Any antimicrobial treatment (%) | 1436 (86.0) | 1287 (85.7) | 149 (88.7) | 0.353 |
| Aminopenicillin (%) | 208 (12.5) | 195 (13.0) | 13 (7.7) | 0.067 |
| Other penicillins (%) | 40 (2.4) | 39 (2.6) | 1 (0.6) | 0.179 |
| Extended-spectrum penicillins (piperacillin-tazobactam) (%) | 683 (40.9) | 599 (39.9) | 84 (50.0) | 0.015 |
| 1st Gen cephalosporins (%) | 79 (4.7) | 76 (5.1) | 3 (1.8) | 0.088 |
| 2nd Gen cephalosporins (%) | 56 (3.4) | 50 (3.3) | 6 (3.6) | 1.000 |
| 3rd Gen cephalosporins (%) | 130 (7.8) | 122 (8.1) | 8 (4.8) | 0.164 |
| 4th Gen cephalosporins (%) | 169 (10.1) | 152 (10.1) | 17 (10.1) | 1.000 |
| 5th Gen cephalosporins (%) | 3 (0.2) | 3 (0.2) | 0 (0.0) | 1.000 |
| Carbapenems (%) | 603 (36.1) | 530 (35.3) | 73 (43.5) | 0.046 |
| Fluoroquinolones (%) | 96 (5.8) | 89 (5.9) | 7 (4.2) | 0.450 |
| Aminoglycosides (%) | 129 (7.7) | 121 (8.1) | 8 (4.8) | 0.172 |
| Macrolides (%) | 214 (12.8) | 189 (12.6) | 25 (14.9) | 0.472 |
| Clindamycin (%) | 63 (3.8) | 56 (3.7) | 7 (4.2) | 0.946 |
| Tetracyclines (%) | 31 (1.9) | 26 (1.7) | 5 (3.0) | 0.406 |
| Sulfonamides (%) | 112 (6.7) | 100 (6.7) | 12 (7.1) | 0.941 |
| Glycopeptides (%) | 192 (11.5) | 171 (11.4) | 21 (12.5) | 0.765 |
| Oxazolidinones (%) | 399 (23.9) | 352 (23.5) | 47 (28.0) | 0.227 |
| Rifamycins (%) | 2 (0.1) | 2 (0.1) | 0 (0.0) | 1.000 |
| Nitroimidazoles (%) | 112 (6.7) | 101 (6.7) | 11 (6.5) | 1.000 |
| Aztreonam (%) | 67 (4.0) | 61 (4.1) | 6 (3.6) | 0.919 |
| Polymyxins (%) | 21 (1.3) | 17 (1.1) | 4 (2.4) | 0.312 |
| Fosfomycin (%) | 161 (9.6) | 135 (9.0) | 26 (15.5) | 0.010 |
| Others (%) | 71 (4.3) | 61 (4.1) | 10 (6.0) | 0.343 |

**Supplementary Table 7** Outcomes in the sensitivity analysis of considering repeated common commensal isolates as clinically relevant

| **Group** | **N** | **Unadjusted Risk (95% CI)** | **Unadjusted RD (95% CI)** | **Unadjusted p** | **Adjusted Risk (95% CI)** | **Adjusted RD (95% CI)** | **Adjusted p** |
| --- | --- | --- | --- | --- | --- | --- | --- |
| **Total sample size** | | | | | | | |
| Monomicrobial | 1859 |  |  |  |  |  |  |
| Polymicrobial | 258 |  |  |  |  |  |  |
| **Death at 90 days** | | | | | | | |
| Monomicrobial | 573 | 30.8% (28.7–32.9%) |  |  | 30.8% (28.9–32.7%) |  |  |
| Polymicrobial | 88 | 34.1% (28.3–39.9%) | 3.29% (-2.87–9.44%) | 0.295 | 35.0% (29.7–40.4%) | 4.25% (-1.41–9.9%) | 0.141 |
| **Death at 30 days** | | | | | | | |
| Monomicrobial | 324 | 17.4% (15.7–19.2%) |  |  | 17.5% (16.0–19.0%) |  |  |
| Polymicrobial | 43 | 16.7% (12.1–21.2%) | -0.76% (-5.63–4.1%) | 0.759 | 17.0% (13.1–20.9%) | -0.48% (-4.68–3.73%) | 0.824 |
| **Microbiologic failure (any pathogen)** | | | | | | | |
| Monomicrobial | 207 | 11.1% (9.7–12.6%) |  |  | 11.1% (9.7–12.5%) |  |  |
| Polymicrobial | 32 | 12.4% (8.4–16.4%) | 1.27% (-3–5.54%) | 0.56 | 12.5% (8.5–16.5%) | 1.43% (-2.8–5.66%) | 0.508 |
| **Microbiologic failure (same pathogen)** | | | | | | | |
| Monomicrobial | 138 | 7.4% (6.2–8.6%) |  |  | 7.3% (6.2–8.5%) |  |  |
| Polymicrobial | 27 | 10.5% (6.7–14.2%) | 3.04% (-0.88–6.96%) | 0.128 | 10.6% (6.8–14.5%) | 3.29% (-0.73–7.31%) | 0.109 |
| **New onset ECMO at 30d** | | | | | | | |
| Monomicrobial | 66 | 3.55% (2.71–4.39%) |  |  | 3.56% (2.8–4.32%) |  |  |
| Polymicrobial | 8 | 3.1% (0.99–5.22%) | -0.45% (-2.73–1.83%) | 0.699 | 3.38% (1.41–5.35%) | -0.18% (-2.29–1.93%) | 0.868 |
| **New onset ARDS at 7d** | | | | | | | |
| Monomicrobial | 328 | 17.6% (15.9–19.4%) |  |  | 17.5% (15.9–19.1%) |  |  |
| Polymicrobial | 37 | 14.3% (10.1–18.6%) | -3.3% (-7.92–1.31%) | 0.161 | 17.5% (13.69–21.3%) | 0% (-3.81–3.81%) | 1 |
| **Fever at 7d** | | | | | | | |
| Monomicrobial | 344 | 18.5% (16.7–20.3%) |  |  | 18.6% (17.1–20.1%) |  |  |
| Polymicrobial | 49 | 19.0 % (14.2–23.8%) | 0.49% (-4.61–5.59%) | 0.851 | 16.4% (12.6–20.2%) | -2.19% (-6.21–1.83%) | 0.285 |
| **Length of ICU stay (days after indexculture)** | | | | | | | |
| Monomicrobial |  | 20.8 (19.8–21.9) |  |  | 20.8 (19.8–21.9) |  |  |
| Polymicrobial |  | 20.8 (17.9–23.8) | 0 (-3.12–3.11) | 0.999 | 20.6 (18.1–23.1) | -0.24 (-2.97–2.49) | 0.865 |
| **Length of hospital stay (days after indexculture)** | | | | | | | |
| Monomicrobial |  | 46.3 (42.9–49.7) |  |  | 46.4 (43.0–49.8) |  |  |
| Polymicrobial |  | 42.8 (33.6–52.0) | -3.51 (-13.3–6.29) | 0.483 | 40.8 (36.0–45.6) | -5.57 (-11.39–0.24) | 0.06 |

# **Supplementary** **Figures**

**
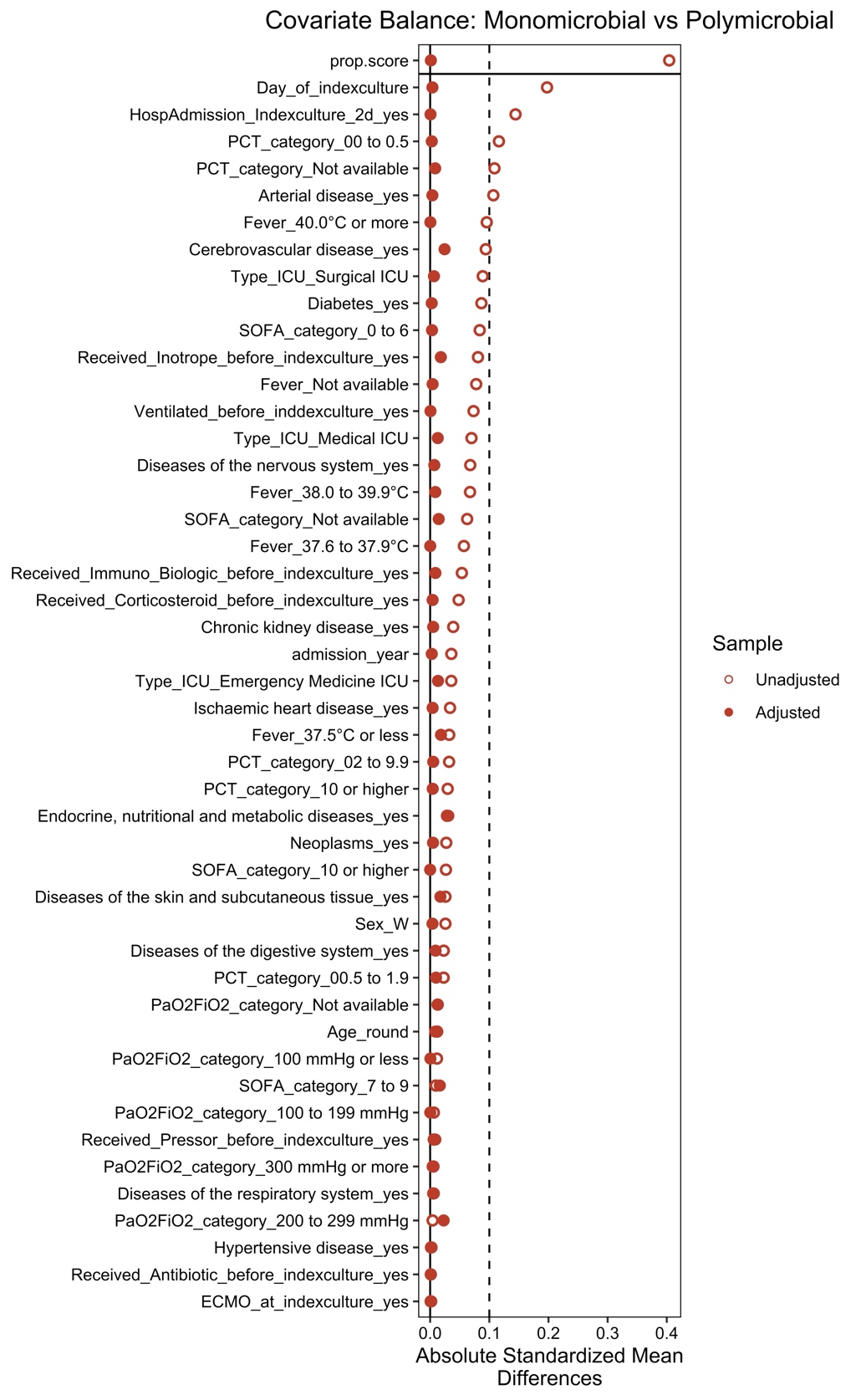
**

**Supplementary Figure 1** Assessment of covariate balance before (empty circles) and after (full circles) inversive probability weighting in the overall cohort.

The vertical dashed lines indicate the usual threshold for acceptable covariate balance, which corresponds to an absolute standardized mean difference (SMD) of 0.1.

**
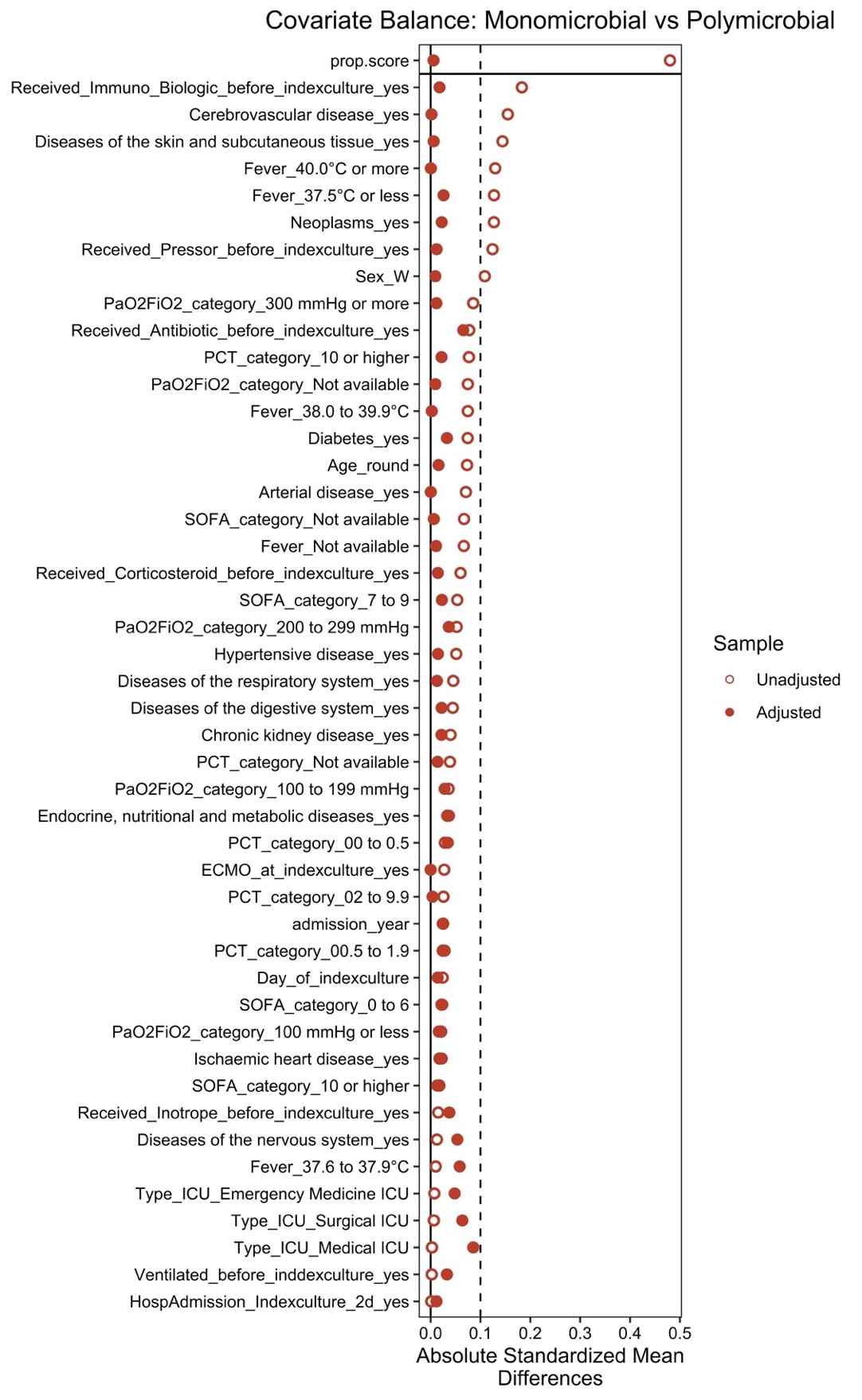
**

**Supplementary Figure 2** Assessment of covariate balance before (empty circles) and after (full circles) inversive probability weighting in the common commensal-free cohort.

The vertical dashed lines indicate the usual threshold for acceptable covariate balance, which corresponds to an absolute standardized mean difference (SMD) of 0.1.


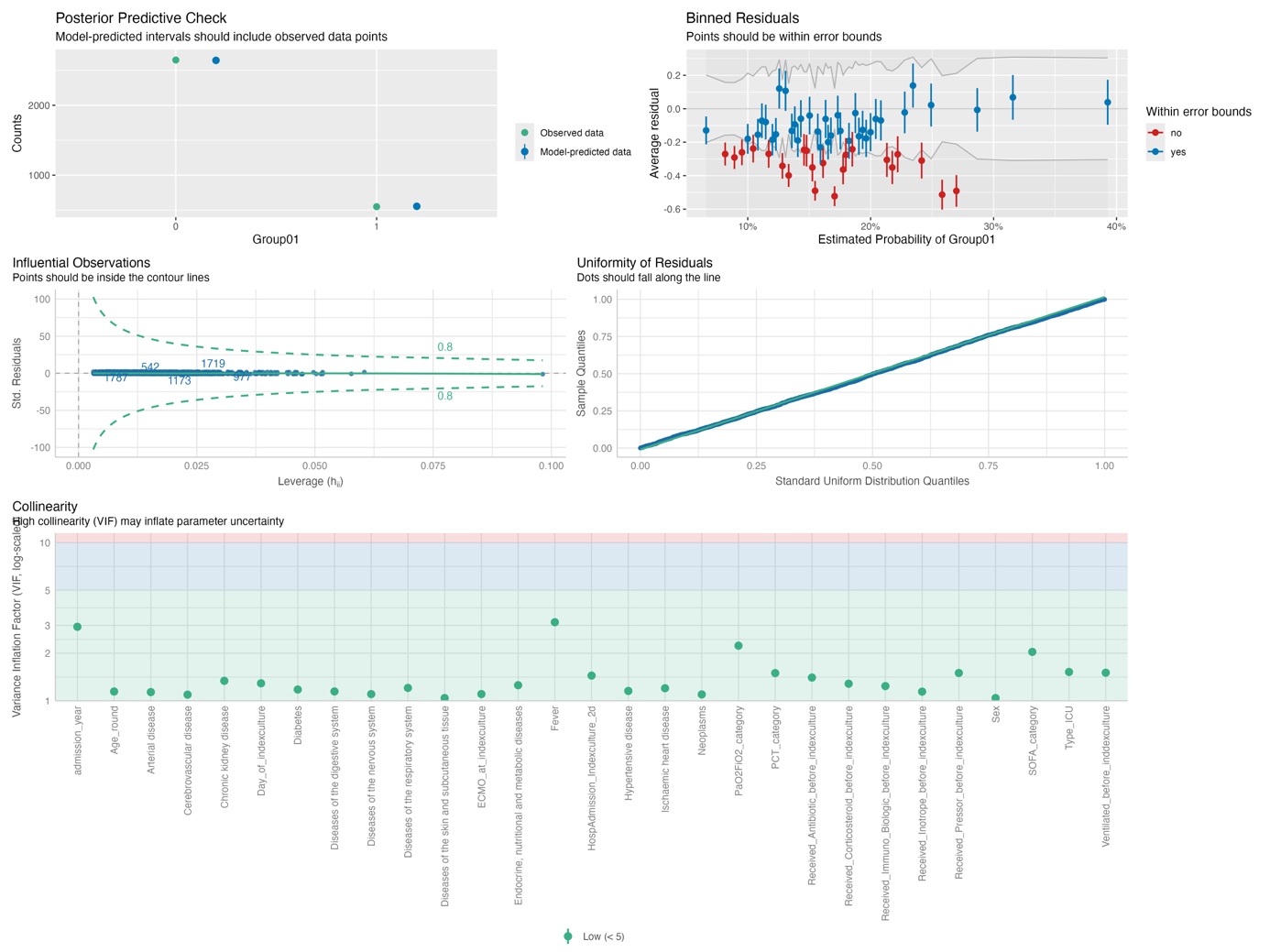


**Supplementary Figure 3** Diagnostic plots of the generalized linear model used for the overall cohort


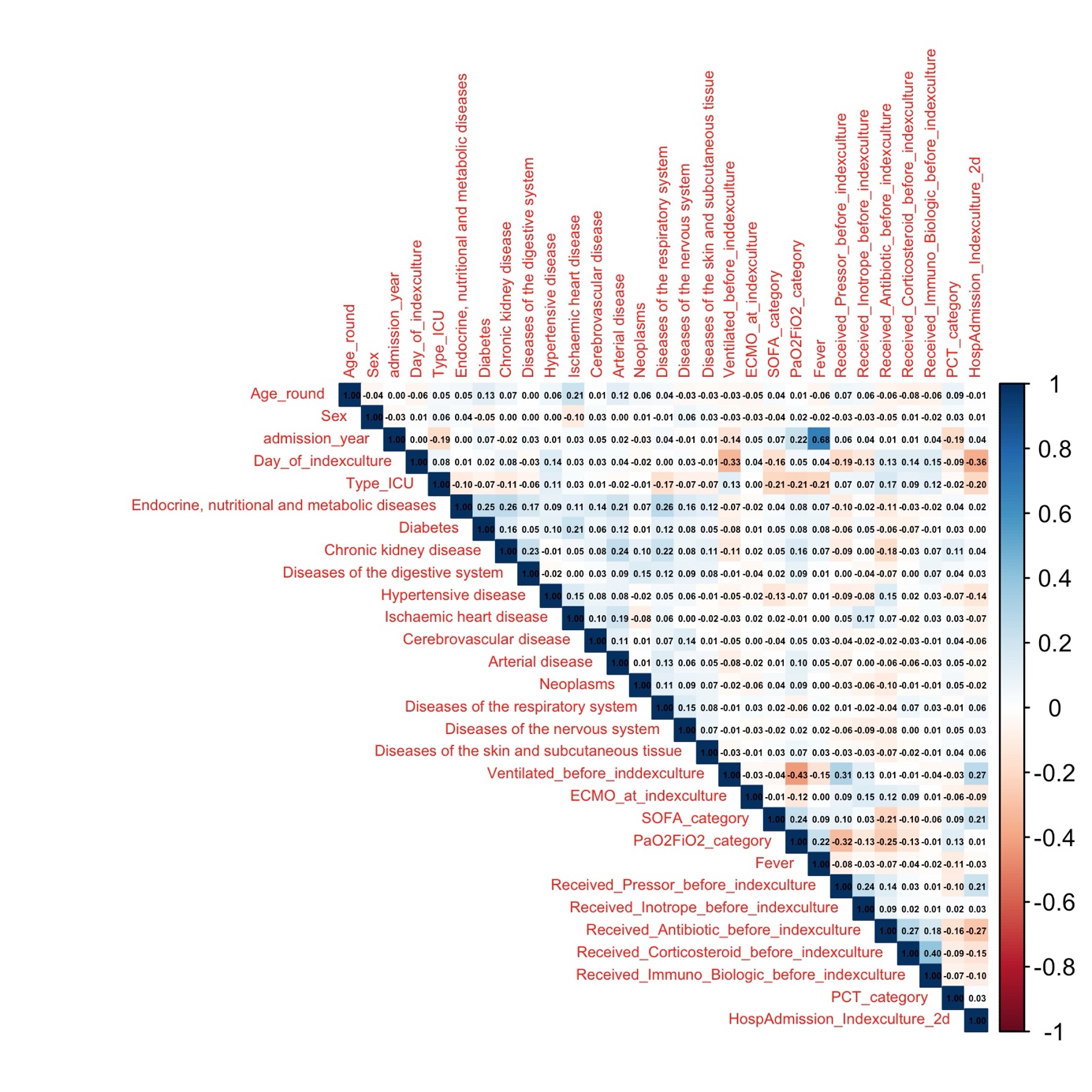


**Supplementary Figure 4** Multicollinearity of covariates used in the weighting and the generalized linear model for the overall cohort

**
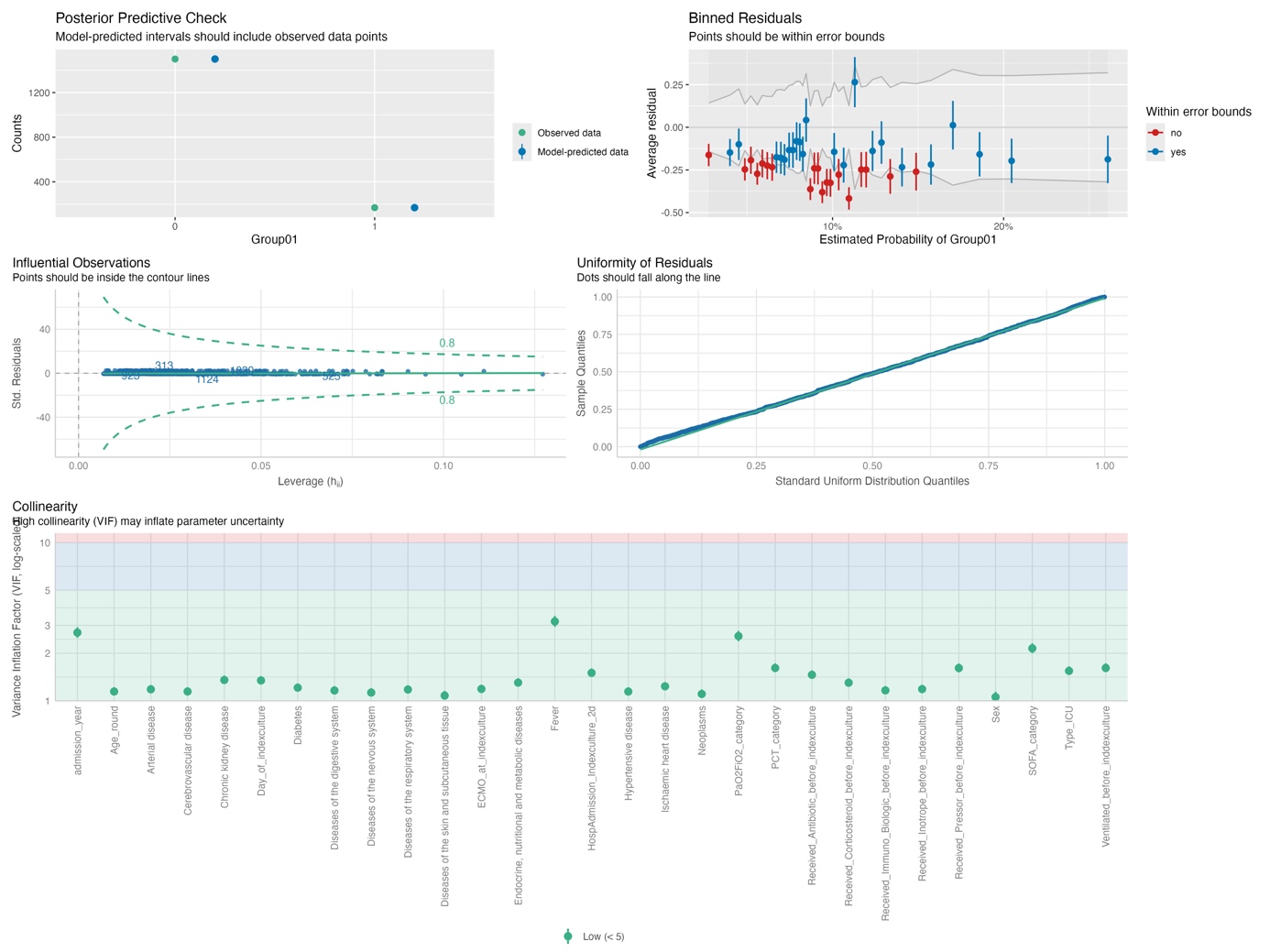
**

**Supplementary Figure 5** Diagnostic plots of the generalized linear model used for the common commensal-free cohort

**
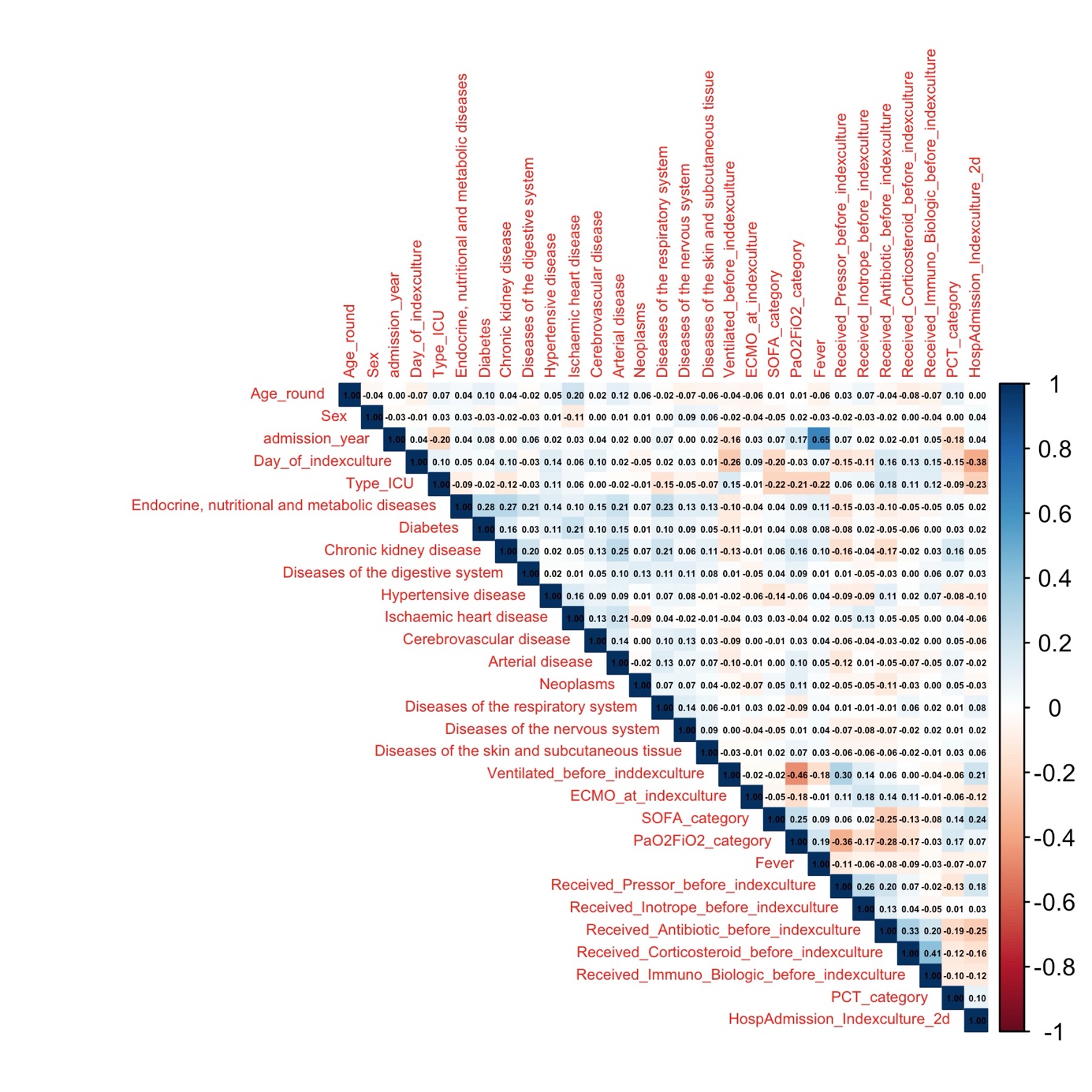
**

**Supplementary Figure 6** Multicollinearity of covariates used in the weighting and the generalized linear model for the common-commensal-free cohort

**Supplementary Figure 6** Exploratory analysis of outcomes by type of polymicrobial infection.
